# *Spem2*, a novel testis-enriched gene, is required for spermiogenesis and fertilization in mice

**DOI:** 10.1007/s00018-024-05147-w

**Published:** 2024-02-29

**Authors:** Chaojie Li, Chunling Shen, Wenfeng Xiong, Haoyang Ge, Yan Shen, Jun Chi, Hongxin Zhang, Lingyun Tang, Shunyuan Lu, Jinjin Wang, Jian Fei, Zhugang Wang

**Affiliations:** 1 State Key Laboratory of Medical Genomics, Research Center for Experimental Medicine, Rui-Jin Hospital affiliated to Shanghai Jiao Tong University School of Medicine, School of Life Sciences and Biotechnology, Shanghai Jiao Tong University, Shanghai, 200025 China; 2 Shanghai Engineering and Technology Research Center for Model Animals, Shanghai Model Organisms Center, Inc, Shanghai, 201203 China

**Keywords:** Male reproduction, Cytoplasmic droplet, Spermatogenesis, Cytoplasm removal, Gene knockout

## Abstract

Spermiogenesis is considered to be crucial for the production of haploid spermatozoa with normal morphology, structure and function, but the mechanisms underlying this process remain largely unclear. Here, we demonstrate that SPEM family member 2 (*Spem2*), as a novel testis-enriched gene, is essential for spermiogenesis and male fertility. *Spem2* is predominantly expressed in the haploid male germ cells and is highly conserved across mammals. Mice deficient for *Spem2* develop male infertility associated with spermiogenesis impairment. Specifically, the insufficient sperm individualization, failure of excess cytoplasm shedding, and defects in acrosome formation are evident in *Spem2*-null sperm. Sperm counts and motility are also significantly reduced compared to controls. In vivo fertilization assays have shown that *Spem2*-null sperm are unable to fertilize oocytes, possibly due to their impaired ability to migrate from the uterus into the oviduct. However, the infertility of *Spem2*^*−/−*^ males cannot be rescued by in vitro fertilization, suggesting that defective sperm–egg interaction may also be a contributing factor. Furthermore, SPEM2 is detected to interact with ZPBP, PRSS21, PRSS54, PRSS55, ADAM2 and ADAM3 and is also required for their processing and maturation in epididymal sperm. Our findings establish SPEM2 as an essential regulator of spermiogenesis and fertilization in mice, possibly in mammals including humans. Understanding the molecular role of SPEM2 could provide new insights into future therapeutic treatment of human male infertility and development of non-hormonal male contraceptives.

**Supplementary Information:**

The online version contains supplementary material available at 10.1007/s00018-024-05147-w.

## Introduction

Mammalian male reproductive ability depends on successful production of haploid spermatozoa with intact paternal genome and normal morphology, structure and function. The process of sperm production in the testis is called spermatogenesis, which can be divided into three different phases [1]: first, spermatogonia self-renew, proliferate and differentiate into primary spermatocytes; secondly, primary spermatocytes undergo meiosis to form round spermatids; and finally, round spermatids transform to elongated spermatids and tadpole-like spermatozoa with complete head and tail structure, which is called spermiogenesis. Spermiogenesis is the longest and most complex phase of spermatogenesis, during which the haploid spermatids undergo dramatic morphological changes and a complex structural reorganization mainly including acrosomal and flagellar development, nuclear elongation, chromatin condensation, mitochondrial rearrangement and elimination of excess cytoplasm. The occurrence of these unique biological events depends on the regulatory roles of testis-enriched genes. Since most of the genes and biological processes involved in spermiogenesis are highly conserved between mice and humans, the mice are considered to be a quite suitable model organism for studying mammalian spermiogenesis. Although great progress has been made in the studies on genetic basis of spermiogenesis by using gene-modified mouse models [2], the exact molecular mechanisms underlying this process remain largely unknown.

The SPEM family consists of three members (SPEM1, SPEM2 and SPEM3), all of which contain no known functional domains and are highly conserved throughout evolution. The mouse ENCODE transcriptome [3] and the human normal tissue RNA-Seq data [4] show that they are all highly enriched in the testicular tissue of both humans and mice, implying that these three proteins may play important physiological roles in male reproduction. In accordance with this, Zheng et al. had characterized SPEM1 as a key regulatory factor responsible for the proper cytoplasm removal in late spermiogenesis [5]. Lack of *Spem1* in mice causes retention of cytoplasmic remnants on the head and neck region, and the retained cytoplasmic remnants obstruct the straightening and stretching of sperm head and neck, leading to sperm deformation and male infertility. Recently it was found that triptonide, a natural compound purified from the Chinese herb *Tripterygium Wilfordii Hook F*, displays reversible male contraceptive effects in both mice and monkeys [6]. Triptonide appears to target junction plakoglobin and disrupts its interactions with SPEM1 during spermiogenesis, indicating SPEM1 may be an ideal non-hormonal contraceptive target. However, no information about the expression and function of the other two members SPEM2 and SPEM3 has been reported yet. Studies on the molecular action of SPEM family members will help us gain more insight into the genetic control of cytoplasm removal during spermiogenesis.

In this study, we focus on the role of SPEM2 in male reproduction. Human *SPEM2*, also known as C17orf74, is located in chromosome 17p13.1 and has 3 exons encoding a 57 kDa protein. Mouse *Spem2* is situated in chromosome 11B3 and is also composed of 3 exons spanning approximately 2 kb. To define the physiological function of *Spem2* in vivo, we generated knockout (KO) mice deficient in this gene. The *Spem2*-KO male mice are completely infertile due to spermiogenic defects. Loss of *Spem2* gene expression leads to failure of excess cytoplasm shedding from the head and neck region of epididymal sperm, which closely resembles the sperm phenotype caused by *Spem1* deficiency. Interestingly, *Spem2*-KO mice develop some unique phenotypes not seen in *Spem1* mutant mice: abnormal acrosome formation, defective chromatin structure, and impaired sperm individualization. Functionally, lack of *Spem2* impairs the fertilization process, which is linked to the down-regulation of ZPBP, PRSS21, PRSS54, PRSS55, ADAM2, and ADAM3 in cauda epididymal sperm. Collectively, these results demonstrate that SPEM2 is involved in the regulation of spermiogenesis and is required for male mouse fertility.

## Results

### *Spem2* is a conserved testis*-*enriched gene

Alignment analyses of the mouse, rat, rabbit, pig, chimpanzee and human SPEM2 orthologs reveal that they are highly conserved during evolution (Supplementary Fig. 1A). The six SPEM2 orthologs exhibit significant sequence identity (62.1–97.6%) (Supplementary Fig. 1B). The mouse SPEM2 shared 64.3% of its amino acids with the human SPEM2. The 80 amino acid residues within the center region and the last 62 amino acid residues at the carboxyl termini of the 6 orthologous proteins are almost identical, suggesting that conserved functional domains may exist in these regions. However, our search using the InterPro [7], which provides an integrative classification of protein sequences into families and identifies functionally important domains and conserved sites, failed to recognize any known functional domains in these proteins. A phylogenetic analysis with 12 mammalian species demonstrates that the mouse SPEM2 is closely related to other mammalian counterparts (Supplementary Fig. 1C), and we also did not find orthologs for this gene in a search of other lower vertebrate genomic databases including those of the fly, zebrafish, and chicken, suggesting that this protein may belong to the mammalian kingdom. Highly conserved sequences across mammalian species imply that SPEM2 may play an important physiological role.

**Fig. 1:**
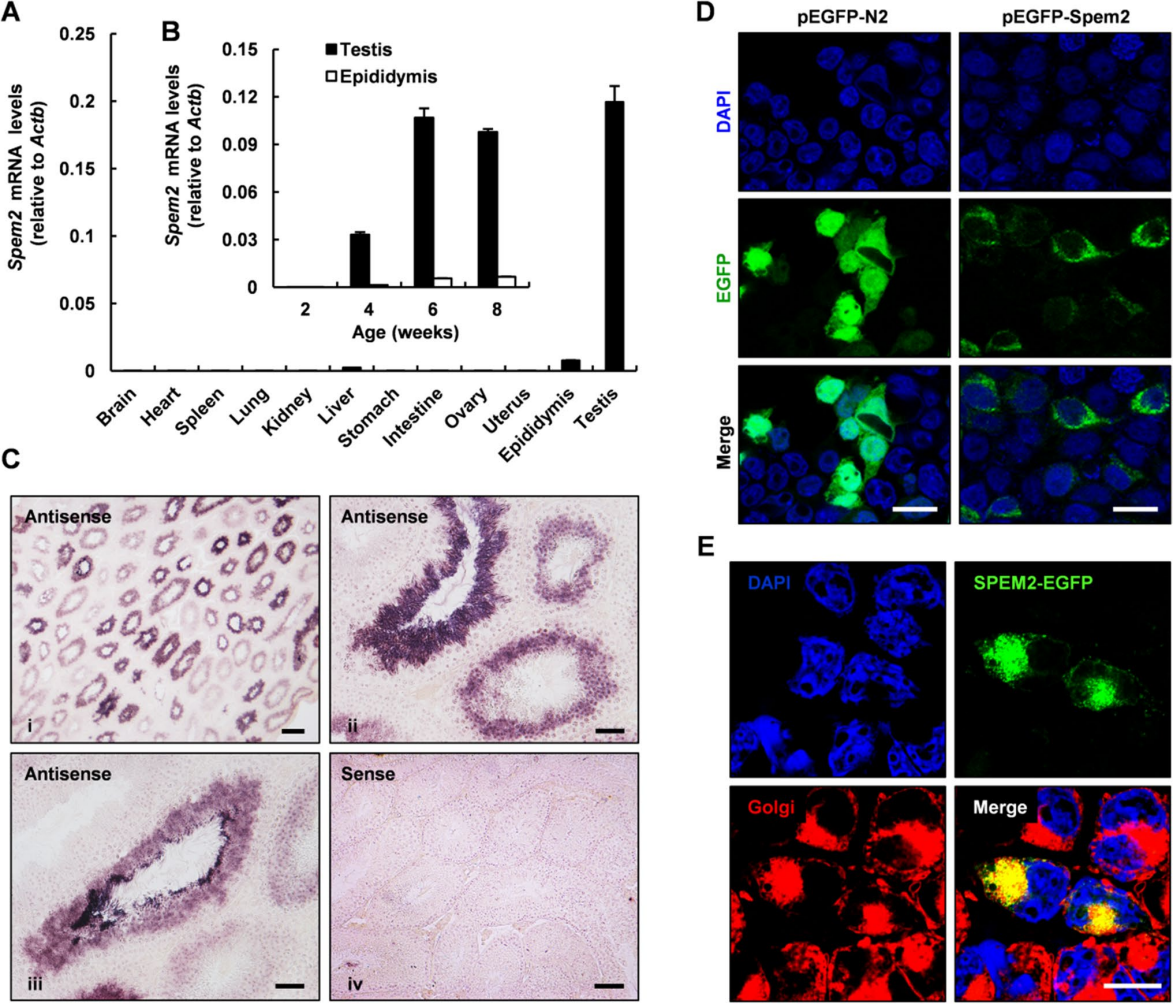
Spatiotemporal expression pattern of *Spem2*. **A** RT-qPCR analysis of *Spem2* mRNA expression in 12 mouse tissues. *Actb* was used as the control. Bar chart shows mean ± SEM values from three independent experiments. **B** Expression of *Spem2* in postnatal mouse testis and epididymis detected by RT-qPCR. *Actb* was used as the control. Bar chart shows mean ± SEM values from three independent experiments. **C** Localization of *Spem2* mRNA in mouse testis by in situ hybridization. (i) Lower-magnification image shows that the hybridization signals are confined to the luminal compartment. Scale bar, 200 μm. (ii, iii) Higher magnification images reveal that the hybridization signals are predominantly observed in the haploid spermatids which are located in the inner half-layer of the seminiferous tubules. Scale bar, 50 μm. (iv) No signals were detected when a sense RNA probe was used as a negative control. Scale bar, 100 μm. **D** The localization of SPEM2-EGFP fusion protein in HEK293T cells was examined by laser confocal microscopy. Nuclei were counterstained with DAPI. Scale bar, 20 μm. **E** The fluorescent Golgi-specific probes and SPEM2-EGFP fusion proteins were co-localized to the perinuclear region. Scale bar, 20 μm

To characterize the expression of *Spem2* gene in mice, we performed real time quantitative PCR (RT-qPCR) assay using cDNA samples prepared from various organs including brain, heart, spleen, lung, liver, kidney, stomach, intestine, ovary, uterus, epididymis and testis. *Spem2* mRNA could be detected in testis and epididymis, but not in other tissues, and it was predominantly expressed in the testis (Fig. 1A), indicating that *Spem2* is a testis-enriched gene in mice. Next, the onset of *Spem2* expression in developing mouse testis and epididymis was examined by RT-qPCR. Results showed that *Spem2* mRNA was first detected around 4 weeks after birth and plateaued in adults (Fig. 1B), which is synchronized with the sexual maturation of male mice. In order to further determine the testicular cell types that express *Spem2* mRNA, in situ hybridization was performed on paraffin sections of adult mouse testis. Hybridization signals using antisense RNA probe were confined to the luminal compartment, and no signal was found in Leydig cells (Fig. 1C i). Positive staining for *Spem2* mRNA was predominantly observed in haploid cells which are located in the inner half-layer of the seminiferous tubules, and more intense staining was detected in the late spermatids (Fig. 1C ii–iii). Sense RNA probe, as a negative control, did not detect any signals above the background levels (Fig. 1C iv). Such a specific expression of *Spem2* in the testis implies that it may be a key regulator in spermiogenesis.

In order to investigate the cellular localization of SPEM2 protein in testis, immunostaining of testicular cross-sections was performed with commercially available antibodies but did not yield specific staining. Next, we attempted to study the localization of SPEM2 through in vitro experiments. pEGFP-Spem2 vector was constructed to express a SPEM2-EGFP fusion protein in which EGFP was fused at the C-terminus of the full-length SPEM2 protein. After transient transfection into HEK293T cells, EGFP signals from the cells transfected with pEGFP-Spem2 vectors appeared to be localized exclusively in the perinuclear region (Fig. 1D, right panel). In contrast, the pEGFP-N2 protein as a control was distributed homogeneously in the whole cell (Fig. 1D, left panel). To further identify the perinuclear compartment where SPEM2-EGFP fusion protein was localized, the transfected HEK293T cells were stained with a fluorescent Golgi-specific probe, BODIPY TR C5-ceramide. The fluorescent Golgi-specific probe and SPEM2-EGFP fusion protein were co-localized to the perinuclear region (Fig. 1E). The results demonstrated that recombinant SPEM2 protein was localized specifically to the Golgi apparatus. Several Golgi-associated proteins had been shown to be essential for acrosome formation [8–10]. Likewise, SPEM2 might also be involved in this process.

### *Spem2-*KO male mice are infertile

To study the physiological function of *Spem2*, we generated *Spem2*-KO mice by CRISPR/Cas9 genome editing system. The entire *Spem2* gene, including the 32-bp-long 5′-untranscribed region, exons 1–3 (except for the last 42 bp of exon 3), and 2 introns in between would be targeted in this model (Supplementary Fig. 2A). A 1894-bp deletion in the *Spem2* locus was confirmed by direct sequencing analysis (Supplementary Fig. 2B). The wild-type (wt), heterozygous and homozygous alleles were distinguished by PCR-based genotyping (Supplementary Fig. 2C). The absence of *Spem2* expression was examined by reverse-transcription PCR (RT-PCR) using testicular cDNAs as template (Supplementary Fig. 2D). As a result, we successfully generated *Spem2*-KO mice with a complete inactivation of the *Spem2* gene. To define the effects of *Spem2* deletion on male fertility, adult wt and *Spem2*-KO males (five for each genotype) were mated with wt adult females over a 1-month period. All control males showed normal fertility and produced a total of 115 pups during the 1 month of mating. However, even though KO males showed normal mating behavior with successful ejaculation and vaginal plug formation comparable to controls, none of the females were pregnant after mating with these KO males and did not produce any offspring (Table 1). *Spem2*^−/−^ females displayed normal fertility (Supplementary Fig. 3A), which is consistent with the fact that *Spem2* is a male germ cell-specific gene and is not expressed in females. These results demonstrate that *Spem2* is required for normal male fertility in mice.

**Table 1:** *Spem2* deficiency leads to male infertility in mice

Male mice	Females	Plugs	Litters	Offsprings (M/F)	FCP (%)	FC (%)	Litter size
wt (*n* = 5)	33	21	16	115 (61/54)	63.6	76.2	7
*Spem2*^*−/−*^ (*n* = 5)	48	24	0	0	50	0	0

FCP = (Plugs/Females) × 100%; FC = (Litters/Plugs) × 100%

*FCP* frequency of copulatory plug, *FC* frequency of conception, *M* male, *F* female

### Deletion of *Spem2* in mice leads to spermiogenic defects

**Fig. 2:**
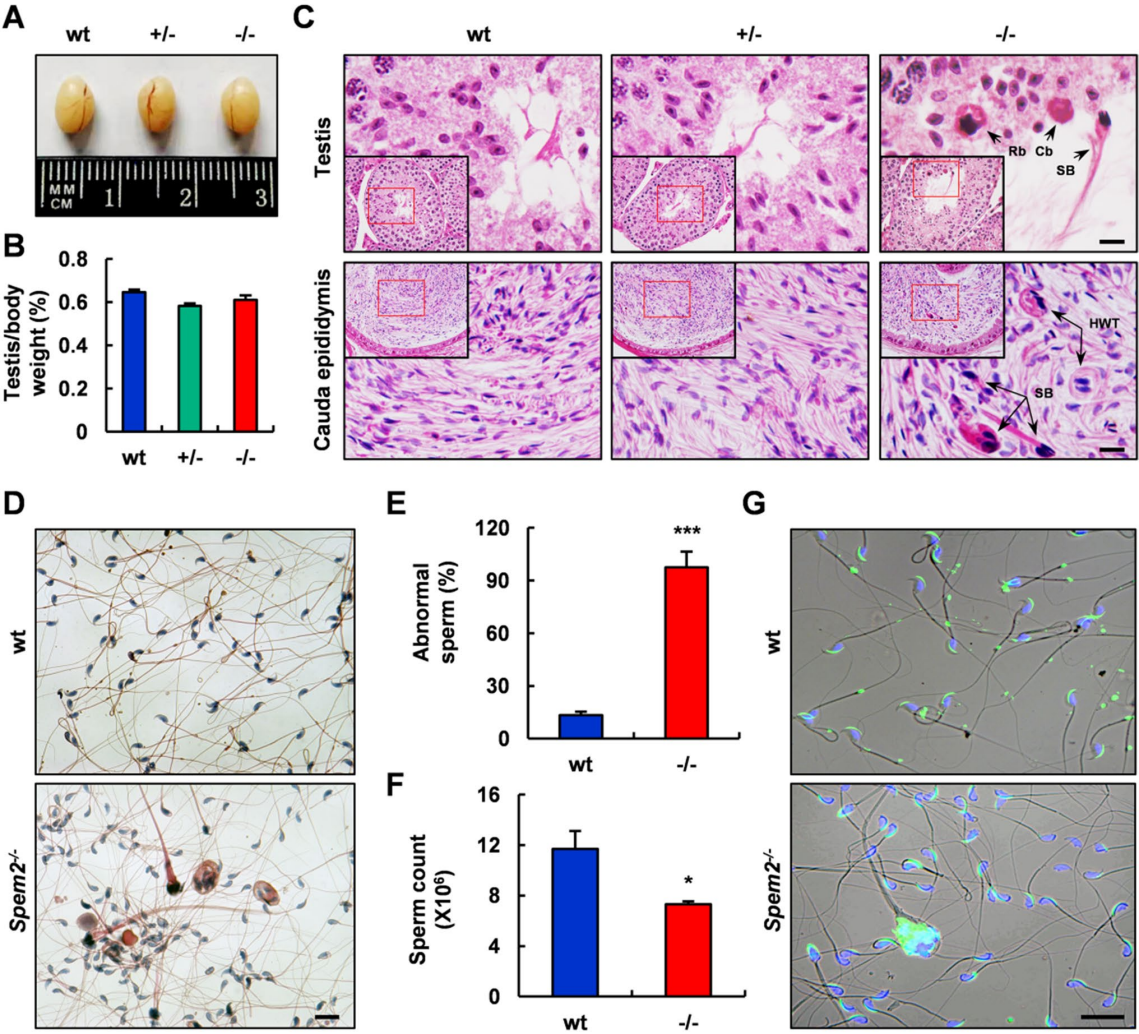
Histological and morphological analyses of testis, cauda epididymis and sperm from wt and *Spem2*^−/−^ mice. **A** Comparison of testicular size of three genotypes of mice. **B** Quantification of the testis/body weight ratio for different genotypes of mice. There were no significant differences. Data bars are mean ± SEM. **C** The histology analysis of the testis and cauda epididymis of wt, heterozygous ( +/−) and homozygous (−/−) mice. Abnormal large residual body (Rb) and cytoplasmic body (Cb) were observed in *Spem2*^−/−^ mice, the sperm bundle (SB) was seen sloughing into the lumen and subsequently appeared in the cauda epididymis. The sperm with heads wrapped by tails (HWT) were also found in *Spem2*^−/−^ mouse cauda epididymis. Scale bar, 10 μm. **D** Morphological analysis of epididymal sperm from wt and *Spem2*^−/−^ mice under light microscope. Scale bar, 20 μm. **E** Quantification of the percentage of abnormal sperm in wt and *Spem2*^−/−^ mice. Data are means ± SEM. ***P < 0.001. **F** Compared with wt mice, the number of epididymal sperm from *Spem2*^−/−^ mice was significantly reduced. Data are means ± SEM. *P < 0.05. **G** PNA staining of epididymal sperm from wt and *Spem2*^−/−^ mice. Scale bar, 20 μm

To unveil the reason for the infertility of *Spem2*^−/−^ mice, we paid close attention to the development and phenotypes of KO males. *Spem2*^−/−^ males did not exhibit any gross abnormalities and survived to adulthood. The serum testosterone levels were also normal in *Spem2*^−/−^ mice (Supplementary Fig. 3B), suggesting that infertility was not caused by hormonal deficiencies. In addition, no distinguishable differences for testicular size were observed among three different genotypes of mice (Fig. 2A), and the testis-to-body weight ratio of KO mice was similar to that of control mice (Fig. 2B). Basic histological assessment of testis cross-sections revealed that in the *Spem2*^−/−^ mice, spermatogonia, spermatocytes and round spermatids appeared normal compared to wt mice (Supplementary Fig. 3C). However, abnormalities began to appear in the elongating spermatids of the mutant mice. Residual bodies were engulfed by Sertoli cells rapidly in control testes, but abnormal large residual body and cytoplasmic body were observed in the KO testes (Fig. 2C, upper panel). Besides, sperm bundles were seen sloughing into the lumen and subsequently appeared in the epididymis, and the sperm with heads wrapped by tails were also found in the mutant mouse epididymis but not in the controls (Fig. 2C, lower panel).

**Fig. 3:**
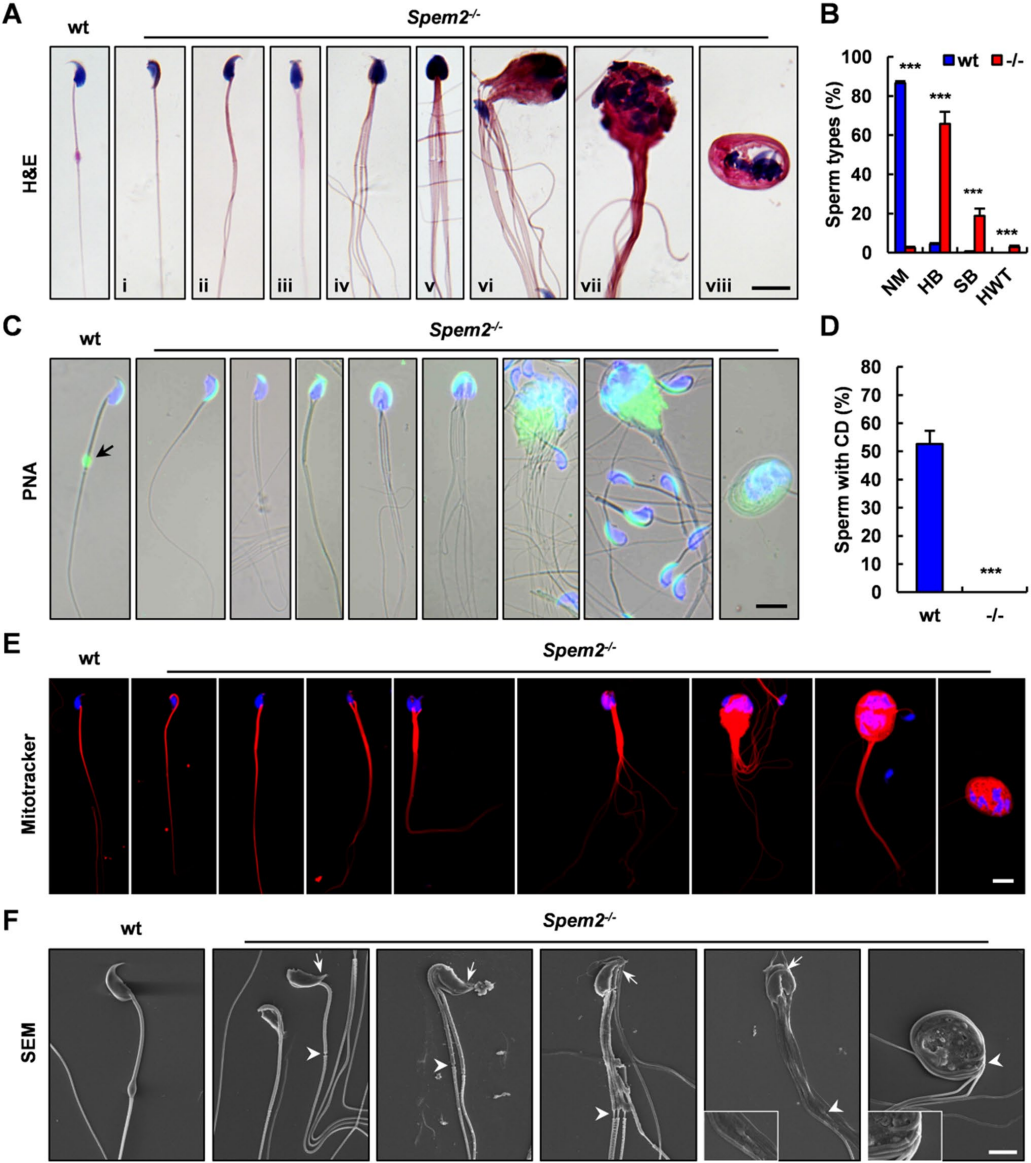
Defects of *Spem2*-null sperm. **A** Representative epididymal sperm of wt and *Spem2*^−/−^ mice examined by H&E staining. Wt sperm showed normal morphology (NM). Multiple abnormalities were observed in the *Spem2*-null sperm, including head bent backward (HB) (i), sperm bundle (SB) (ii-vii), and heads wrapped by tails (HWT) (viii). Scale bar, 10 μm. **B** Quantification of the percentage of different types of sperm in wt and *Spem2*^−/−^ mice. Data are means ± SEM. ***P < 0.001. **C** Representative epididymal sperm of wt and *Spem2*^−/−^ mice examined by PNA staining. Well-developed acrosome and cytoplasmic droplet (CD) (arrow) were present in wt sperm. The *Spem2*-null sperm had abnormal acrosome, and the CD was missing in *Spem2*^−/−^ mice. Scale bar, 10 μm. **D** Proportion analysis of sperm with CD in the wt and *Spem2*^−/−^ mice. Data are means ± SEM. ***P < 0.001. **E** Representative images from MitoTracker Red staining, showing the presence of normal mitochondrial activity but abnormal mitochondrial position in *Spem2*^−/−^ sperm. Scale bar, 10 μm. **F** Representative epididymal sperm of wt and *Spem2*^−/−^ mice examined by SEM. Consistently, different types of abnormal sperm were observed in *Spem2*^−/−^ mice. In addition, reduced curvature at the head tip (arrows) and discontinuous accessory structure in the middle piece (arrowheads) were also seen. Scale bar, 5 μm

Next, we collected epididymal sperm from adult wt and *Spem2*^−/−^ mice for further analysis. In contrast to controls, there were almost no normal morphology sperm in the mutant mice, as examined by H&E staining and light microscopy analysis (Fig. 2D). The percentage of morphologically abnormal sperm from *Spem2*^−/−^ males was calculated to be 97.5%, which was significantly different compared to wt males in which only 13.4% of sperm were of abnormal morphology (Fig. 2E). Furthermore, the number of sperm in cauda epididymis was reduced by 37.6% in *Spem2*^−/−^ mice as compared with wt mice (Fig. 2F). High-power microscopic examination of sperm smears further confirmed that there were various morphological defects in *Spem2*^−/−^ sperm (Fig. 3A). These abnormal sperm could be classified into 3 groups, the sperm with head bent back onto the midpiece (Fig. 3A i), the sperm that were clumped together with cellular remnants along their heads (Fig. 3A ii–vii), and the sperm with tails coiled around their heads (Fig. 3A viii). The statistical analysis revealed the significant higher percentage of these 3 kinds of defective sperm in *Spem2*^−/−^ mice, compared to those from wt mice, respectively (Fig. 3B). To test whether *Spem2* deficiency has any impact on acrosome formation, the acrosome was examined with fluorescein labeled PNA. Compared with acrosomes of wt sperm, abnormal acrosomes were mostly present in the KO sperm (Fig. 2G). A well-formed acrosome was developed over the anterior part of the sperm head in control sperm. However, in mutant sperm, the PNA signals labeling acrosome were defective and showed abnormal acrosome localization (Fig. 3C). Notably, the cytoplasmic droplet (CD) could be detected at the tail of over half of wt sperm, while it was missing in that of *Spem2*^−/−^ sperm by PNA staining (Fig. 3C, D). Moreover, the mitochondrial sheath was disorganized, with mitochondria scattered in varied locations, although the mitochondria retained normal function, as shown by positive staining on MitoTracker assay (Fig. 3E). In addition, the sperm motility of the *Spem2*^−/−^ mice was damaged, with the significantly decreased motility rate compared to that of the wt mice (Supplementary Table 1). Consistently, the structural defects in *Spem2*^−/−^ sperm were evident by scanning electron microscopy (SEM) (Fig. 3F). Moreover, reduced curvature at the head tip and discontinuous accessory structure in the middle piece were observed in *Spem2*^−/−^ sperm by SEM (Fig. 3F). Collectively, these results revealed the presence of multiple defects during spermiogenesis in *Spem2*^−/−^ mice.

### *Spem2*^−/−^ male mice show defects in acrosome development, cytoplasm removal and sperm individualization

**Fig. 4:**
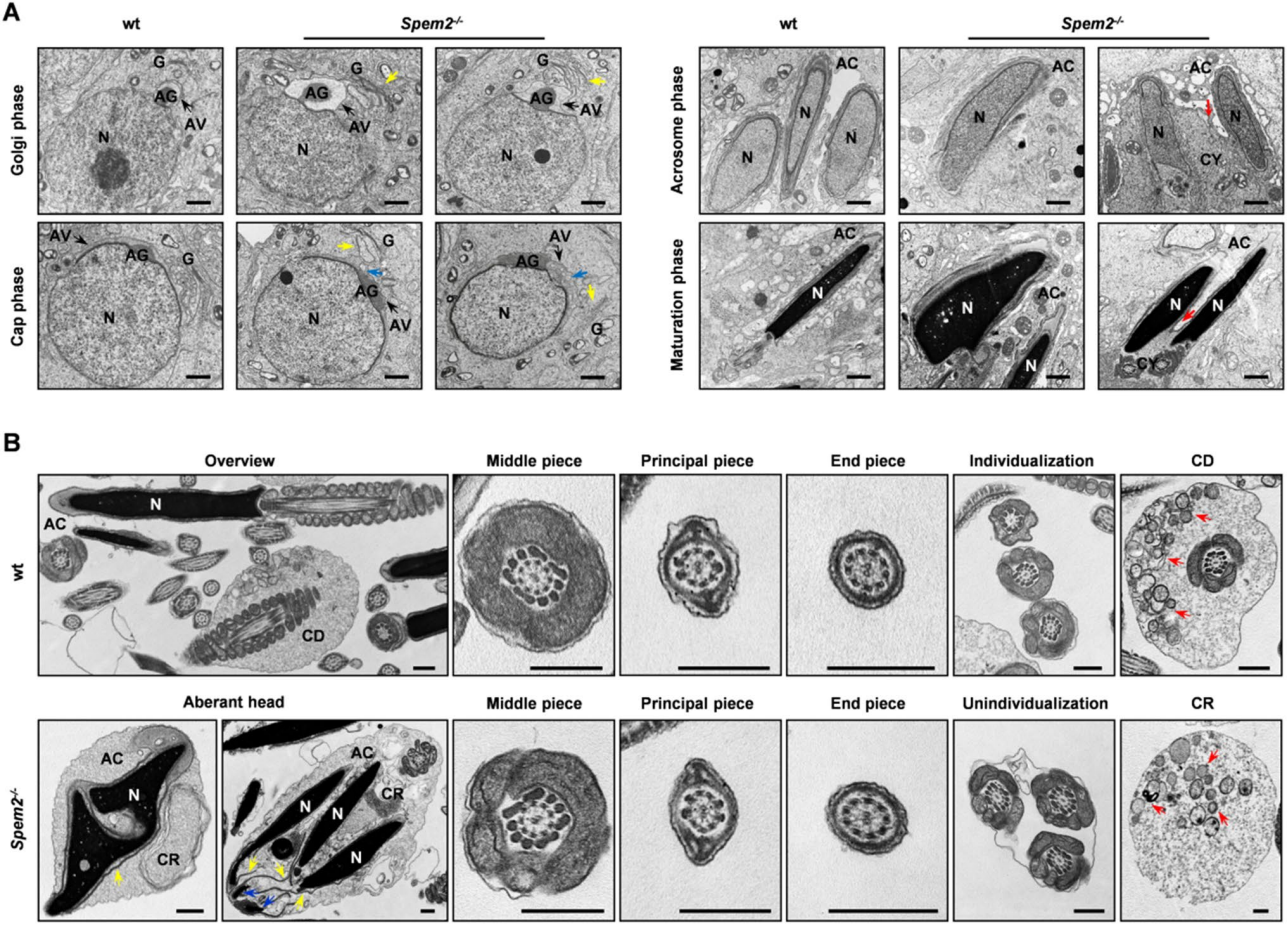
TEM analysis of impaired spermiogenesis in *Spem2*^−/−^ mice. **A** Abnormal acrosome biogenesis in *Spem2*^−/−^ mice was shown by TEM analysis, including morphologically abnormal Golgi apparatus (G) (yellow arrows) and vacuolated (blue arrows) or irregular shaped acrosomal vesicles (AV), which induced abnormal acrosome (AC) morphology in the maturation phase. In addition, irregularly shaped nuclei (N), abundant cytoplasm (CY) and abnormal removal of intercellular bridges (red arrows) were observed in *Spem2*^−/−^ mice. AG, acrosomic granule. Scale bar, 1 μm. **B** Numerous abnormalities in *Spem2*^−/−^ sperm were observed by TEM analysis, including abnormal acrosome and chromatin structure, multiple nuclei, detached acrosome membranes (yellow arrows), extra nuclear chromatin material (blue arrows) and retained cytoplasmic remnant (CR) in the head and impaired sperm individualization. CD, cytoplasmic droplet; Red arrows indicate small membranous vacuoles. Scale bar, 0.5 μm

To further define the nature of the defects during spermiogenesis in *Spem2*^−/−^ mice, transmission electron microscopy (TEM) analysis was performed to analyze the ultrastructures of the spermatids at different developmental steps of spermiogenesis. In the wt mice, a concave Golgi complex formed over the round spermatid nucleus and released proacrosomic granules that fused to form an acrosomal vesicle, with a single, large acrosomal granule that flattened onto the nucleus (Fig. 4A, left panel). Elongating/elongated spermatids had highly condensed nucleus and hook-shaped acrosome tightly attached to the nucleus (Fig. 4A, right panel). However, vacuolated or irregular shaped acrosomal vesicles and morphologically abnormal Golgi apparatus that did not have the typical arch over the nucleus were observed in the round spermatids of *Spem2*^−/−^ mice (Fig. 4A, left panel), which caused abnormal acrosome in the maturation phase. In addition, irregularly shaped nuclei and abundant cytoplasm were distinct in the late spermatids (Fig. 4A, right panel). Furthermore, multiple elongating spermatids or spermatozoa were found wrapped in a single cell membrane, indicating defective removal of intercellular bridges between individual spermatids (Fig. 4A, red arrows). These results suggest that *Spem2* deficiency appears to have disrupted one or more of the steps in spermiation, a process involving remodeling of the spermatid head, shedding of excess cytoplasm, dissolution of intercellular bridges and disengagement of the spermatid from the Sertoli cell [11].

TEM analyses of epididymal sperm demonstrated that all the structural components at the tail were intact in *Spem2*^−/−^ sperm compared with wt sperm (Fig. 4B). Consistent with H&E staining and SEM observation, the same kinds of abnormal sperm were also observed under TEM (Supplementary Fig. 4). For the sperm with bent head, the head and the middle piece of the tail were held together by tissues resembling remnants of the cytoplasm that should have been completely shed off during spermiation. For the aggregated sperm, double/multiple heads and multiple (two or more) flagella were enfolded by residual cytoplasm, suggesting a failure of sperm individualization. Moreover, TEM showed sperm heads with various abnormal ultrastructures, including the abnormal acrosome, defective chromatin structure, detached acrosome membranes (Fig. 4B). In addition, in some sperm, nuclear membranes were disrupted and what appear to be units of chromatin were found outside the boundaries of the nucleus (Fig. 4B, blue arrows). Given that all *Spem2*^−/−^ sperm do not have CDs, we compared the ultrastructure of normal CDs with the cytoplasmic remnants in the *Spem2*-null sperm. The contents in the CDs showed some similar features compared with those of cytoplasmic remnants on the *Spem2*^−/−^ sperm. They were both homogenous and contain many small membranous vacuoles (Fig. 4B, red arrows). Therefore, lack of CDs in the *Spem2*^−/−^ sperm may be caused by impaired detaching and shedding of the cytoplasm from the head region of sperm.

### Retention of cytoplasmic remnants in the head region causes defective acrosome reaction in *Spem2-*null sperm

**Fig. 5:**
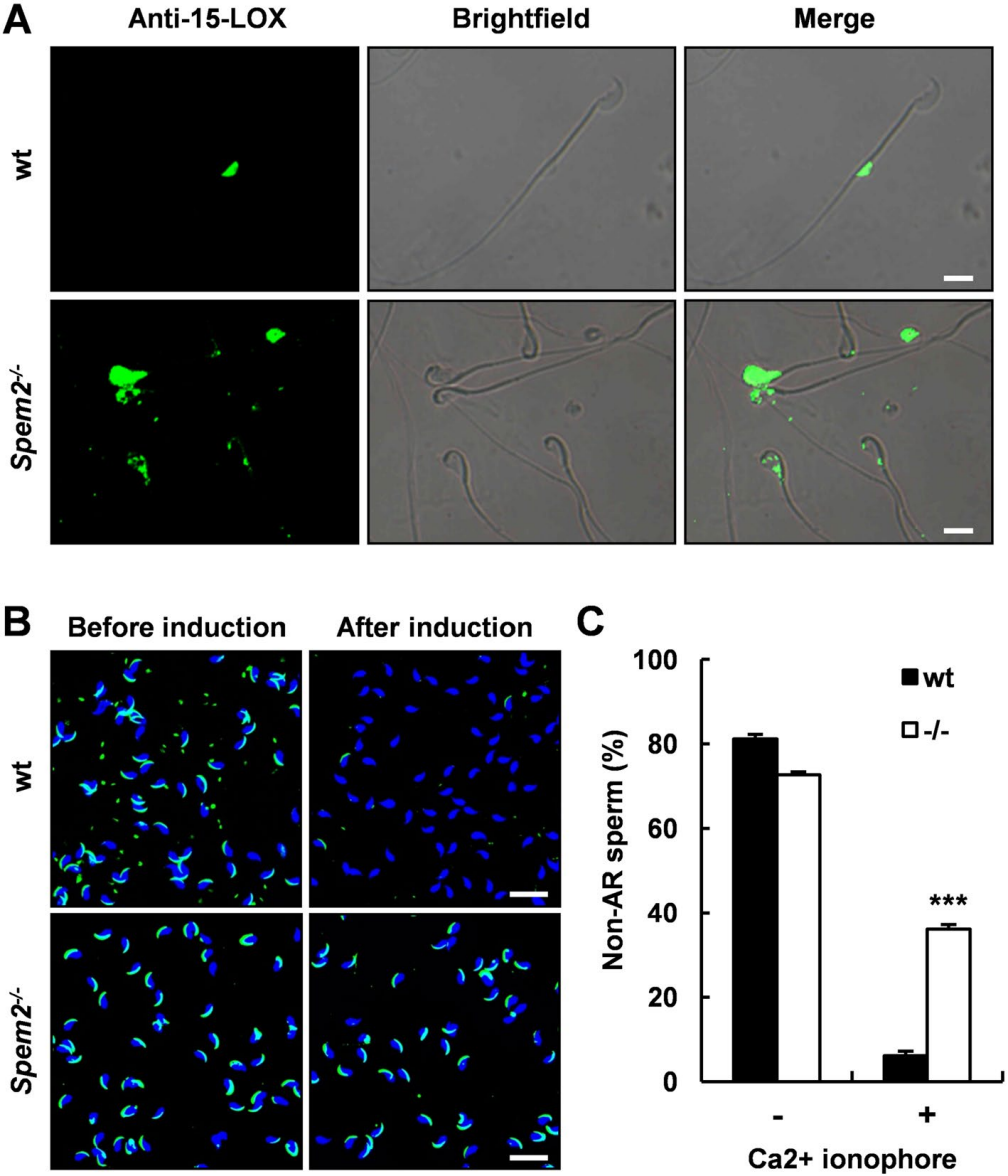
Defective sperm acrosome reaction in *Spem2*^−/−^ mice. **A** Immunofluorescence for the CD marker 15-LOX in sperm from wt and *Spem2*^−/−^ mice. Scale bar, 10 μm. **B** PNA-FITC staining of sperm from wt and *Spem2*^−/−^ mice before and after induction by calcium ionophore A23187. Nuclei were counterstained with DAPI. Representative images were shown. Scale bar, 20 μm. **C** The percentages of non-acrosome-reacted (non-AR) sperm (PNA positive) of the experiment in B. Data are means ± SEM. ***P < 0.001

Considering the ultrastructural similarity between the cytoplasmic remnants on the *Spem2*-null sperm and normal CDs, we speculate that they may have the same origin. The 15-lipoxygenase (15-LOX) which is highly expressed in the cytoplasm of late spermatids has been detected in CDs and is suggested to have a role in the cytoplasm removal [12]. Moreover, many other proteins that are highly expressed in the cytoplasm of late spermatids can also be detected in CDs, suggesting that CDs structurally are derived from the cytoplasm of late spermatids. To determine whether the cytoplasmic remnants on the *Spem2*^−/−^ sperm are also derived from the cytoplasmic components of late spermatids, we performed immunofluorescent detection of 15-LOX in epididymal sperm. As expected, the 15-LOX signal was observed in CDs of wt sperm, whereas it was presenting the head regions where redundant cytoplasms were detained in the *Spem2*^−/−^ sperm (Fig. 5A). These results further support our TEM observations, suggesting that the cytoplasmic remnants in the head regions of *Spem2*-null sperm, like CDs, are originated from the shed cytoplasm of late spermatids.

All mammalian sperm have an intact acrosome and the acrosome reaction is a prerequisite for successful fertilization [13]. During the acrosome reaction, the outer acrosomal membrane fuses with the sperm plasma membrane and hydrolytic enzymes in the acrosome are released to facilitate fertilization. Normally, the outer acrosomal membrane is underlying the sperm plasma membrane and closely adjacent to each other. However, in *Spem2*^−/−^ sperm, the interstice between the plasma membrane and the acrosome membrane was filled with excess cytoplasm (Fig. 4B), which greatly hindered the fusion of the two independent membranes, thereby affecting the occurrence of acrosome reaction. To prove that depletion of *Spem2* affects acrosome function, we induced the acrosome reaction in vitro by adding Ca^2+^ ionophore A23187 to capacitated sperm. The acrosome reaction was then monitored by staining with fluorescently labeled PNA. Since A23187 could induce the release of acrosomal contents, only intact acrosomes could be stained by PNA, while acrosome-reacted sperm could not be stained by PNA [14]. Before induction, the proportion of PNA-positive sperm was comparable between wt and *Spem2*^−/−^ mice (81.1% versus 72.6%, Fig. 5B, left panel; Fig. 5C). Strikingly, post-ionophore treatment, most of the sperm were still stained by PNA in *Spem2*^−/−^ mice, while only 6.2% of sperm were PNA-positive in wt mice (Fig. 5B, right panel; Fig. 5C), suggesting *Spem2*^−/−^ sperm failed to release their acrosomal contents after induction. Hence, retention of redundant cytoplasm in the head region causes defective acrosome reaction in *Spem2*^*−/−*^ sperm.

### In vivo and in vitro fertility of *Spem2-*null sperm

**Fig. 6:**
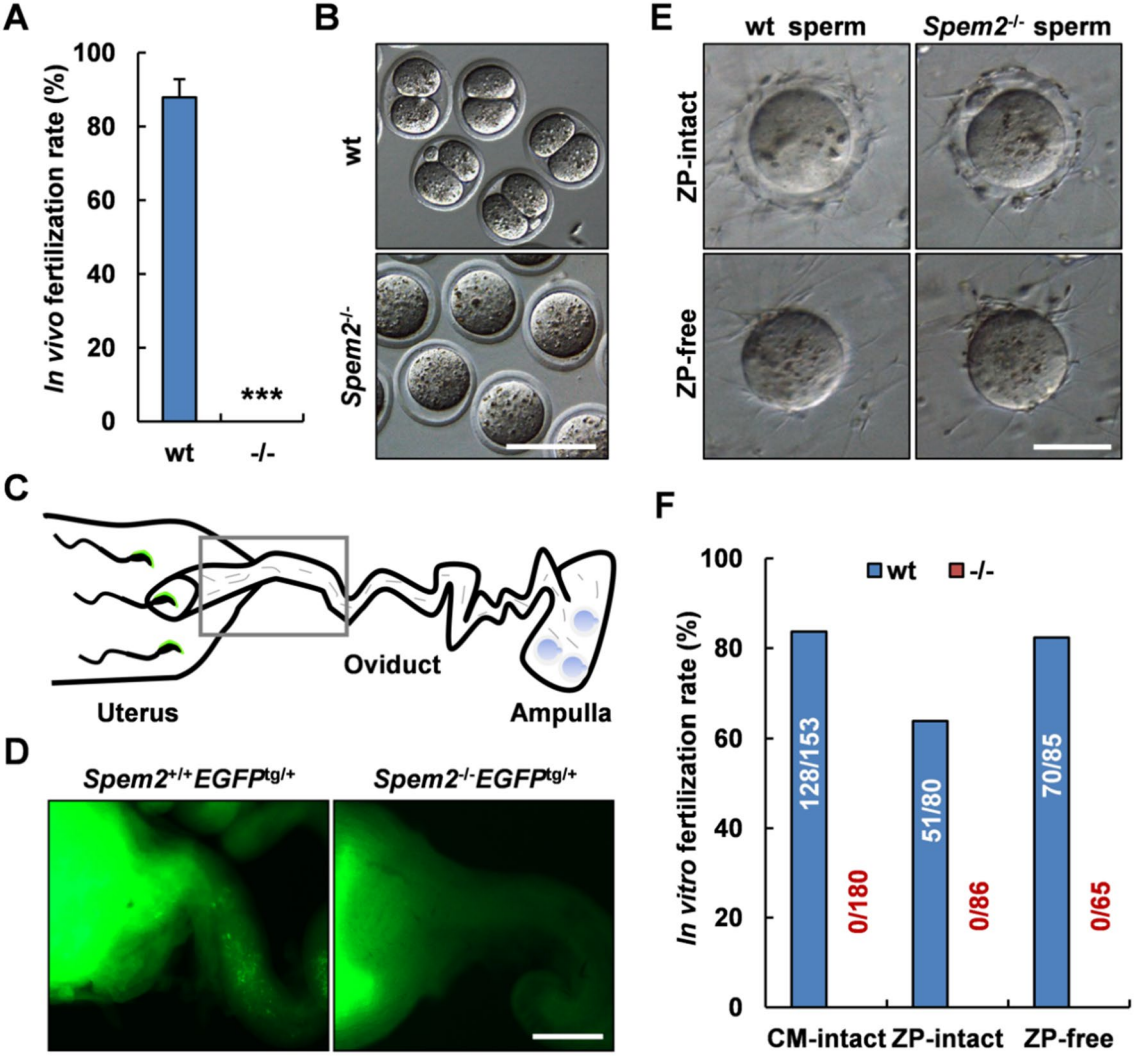
Ablation of *Spem2* in mice leads to fertilization defects. **A** The wt or *Spem2*^−/−^ male mice were mated with the wt females, and oviducts were checked for 2-cell embryos. No *Spem2*^−/−^ mice had 2-cell embryos, compared with 87.9% in the wt mice. Data are means ± SEM. ***P < 0.001. **B** Representative images of the experiment in A. Scale bar, 100 μm. **C** Schematic diagram of EGFP-expressing sperm migration in female genital tract. The boxed area corresponds to the view field in D. **D** Visualization of sperm inside the female reproductive tract 2 to 3 h after observing vaginal plugs. Scale bar, 500 μm. **E** Sperm-egg binding analysis of wt and *Spem2*^*−/−*^ sperm with zona pellucida (ZP)-intact or ZP-free oocytes. Scale bar, 50 μm. **F** In vitro fertilization was performed with cumulus (CM)-intact, ZP-intact and ZP-free oocytes. The numbers marked in or above each column represent the number of oocytes developed to 2-cell stage and the total number of oocytes incubated with sperm at the beginning of the fertilization assays from several experiments

We next analyzed the fertilizing ability of sperm from *Spem2*^−/−^ mice by employing in vivo fertilization experiments. As expected, *Spem2*-null sperm could not fertilize eggs by natural mating (Fig. 6A, B): Sperm from *Spem2*^−/−^ mice yielded no (0%) two-cell embryos, whereas the average fertilization rate using sperm from wt mice was 87.9%, which further confirmed the male infertility phenotype of *Spem2*^−/−^ mice. The findings suggested that the infertility of *Spem2*^−/−^ males was primarily caused by fertilization failure rather than embryonic developmental disabilities.

Considering the array of abnormalities observed with sperm from *Spem2*^−/−^ mice, the inability of *Spem2*^−/−^ sperm to fertilize wt oocytes could be due to multiple reasons, including impaired migration of sperm from uterus into oviduct or a defect in gamete interaction at fertilization. In order to examine the in vivo sperm migration ability, we introduced transgenic mice with EGFP-tagged acrosome [15] to the *Spem2*^−/−^ genetic background to visualize the sperm inside the female reproductive tract (Fig. 6C). When females were mated with *Spem2*^+/+^*EGFP*^tg/+^ males, and the oviducts were excised together with the connective part of the uterus, a large number of sperm were observed in both uterus and oviduct 2 h after coitus (Fig. 6D, left panel). However, when females were mated with *Spem2*^−/−^*EGFP*^tg/+^ males, many sperm were found only in uterus but not in oviduct (Fig. 6D, right panel). This clearly indicates that the *Spem2*^−/−^ sperm lose their oviduct-migrating ability. To overcome this obstacle, we then performed in vitro fertilization and found that *Spem2*^−/−^ sperm could not fertilize cumulus-intact oocytes (Fig. 6F). Intriguingly, removing cumulus cells could not rescue impaired fertilization rates, suggesting that SPEM2 may also be required for the sperm-oocyte interaction. In mammals, this interaction occurs in two steps: Sperm first needs to bind and penetrate the zona pellucida (ZP) before binding to the oocyte plasma membrane, which enables sperm-egg fusion. Notably, *Spem2*-null sperm were unable to fertilize cumulus-free ZP-intact oocytes (Fig. 6F), but these sperm could bind to the ZP (Fig. 6E, upper panel), which is consistent with defective acrosome reaction in *Spem2*^−/−^ mice. Furthermore, even without the ZP, *Spem2*^−/−^ sperm still failed to fertilize the eggs although the sperm bound to the oolemma (Fig. 6E, lower panel; Fig. 6F), implying that the process of sperm-egg fusion was damaged. Under normal conditions, only acrosome-reacted sperm can fuse with the oocytes [16, 17], while *Spem2*^−/−^ sperm seldom underwent the acrosome reaction (Fig. 5B, C), which would greatly hinder this process. Therefore, the fertilization failure was not solely attributed to a defect in sperm migration within the female genital tract, and defective sperm-egg interaction might be a critical factor. Together, these findings demonstrated that SPEM2 is required for sperm fertilizing capacity.

### SPEM2 interacts with ZPBP, PRSS21, PRSS54, PRSS55, ADAM2, and ADAM3, and its absence affects the processing and maturation of these proteins in epididymal sperm

**Fig. 7:**
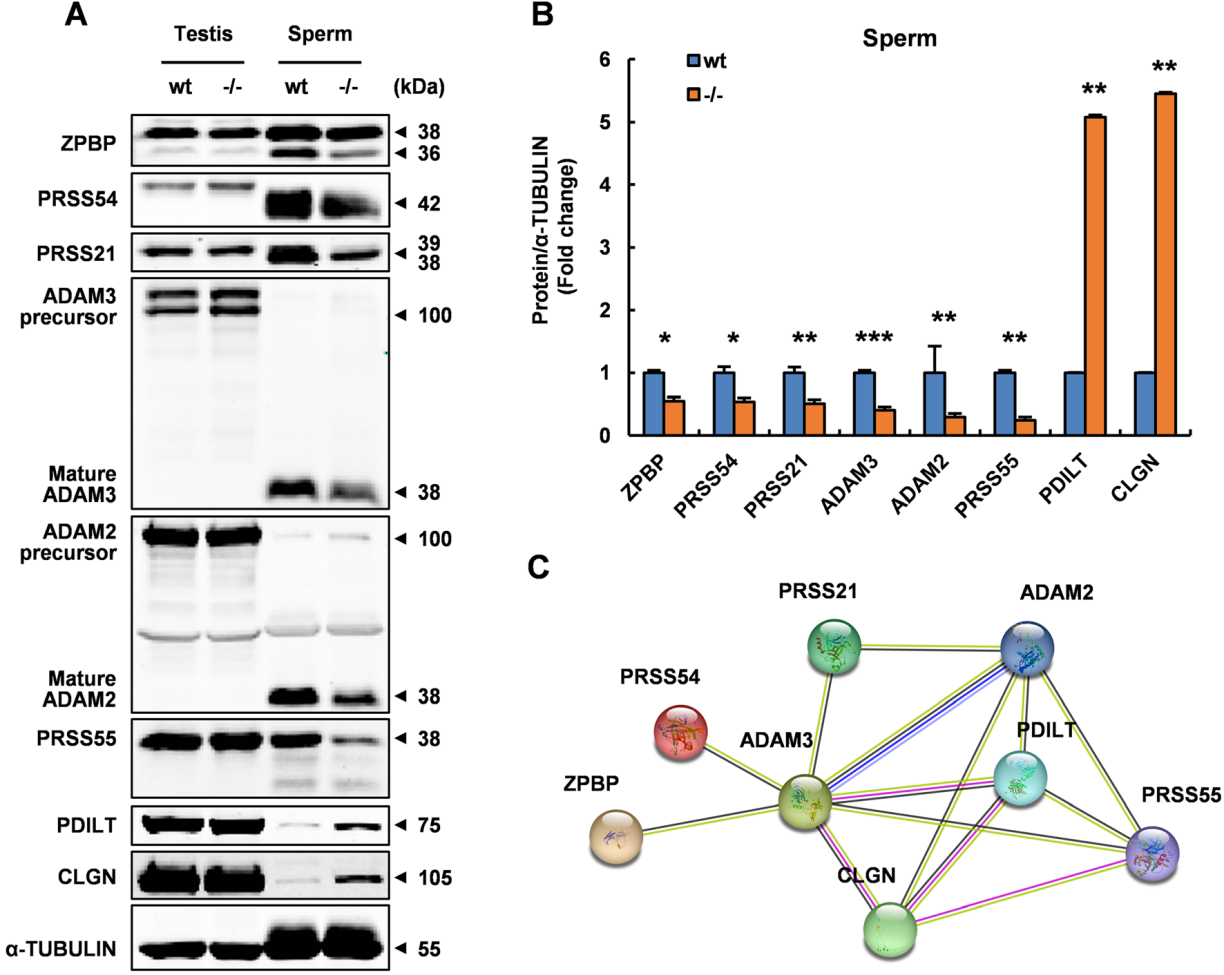
*Spem2* deficiency leads to abnormal expression of ZPBP, PRSS21, PRSS54, PRSS55, ADAM2, ADAM3, CLGN and PDILT in the epididymal sperm. **A** The total lysates of testis and sperm from adult wt and *Spem2*^−/−^ mice were subjected to western blotting analysis using the indicated antibodies. α-TUBULIN was used as a loading control. **B** Western blotting analyses were statistically quantified by densitometry. Data from three independent experiments and are presented as the mean ± SEM. *P < 0.05, **P < 0.01, ***P < 0.001. **C** Interaction network analyses of the dysregulated proteins by STRING

**Fig. 8:**
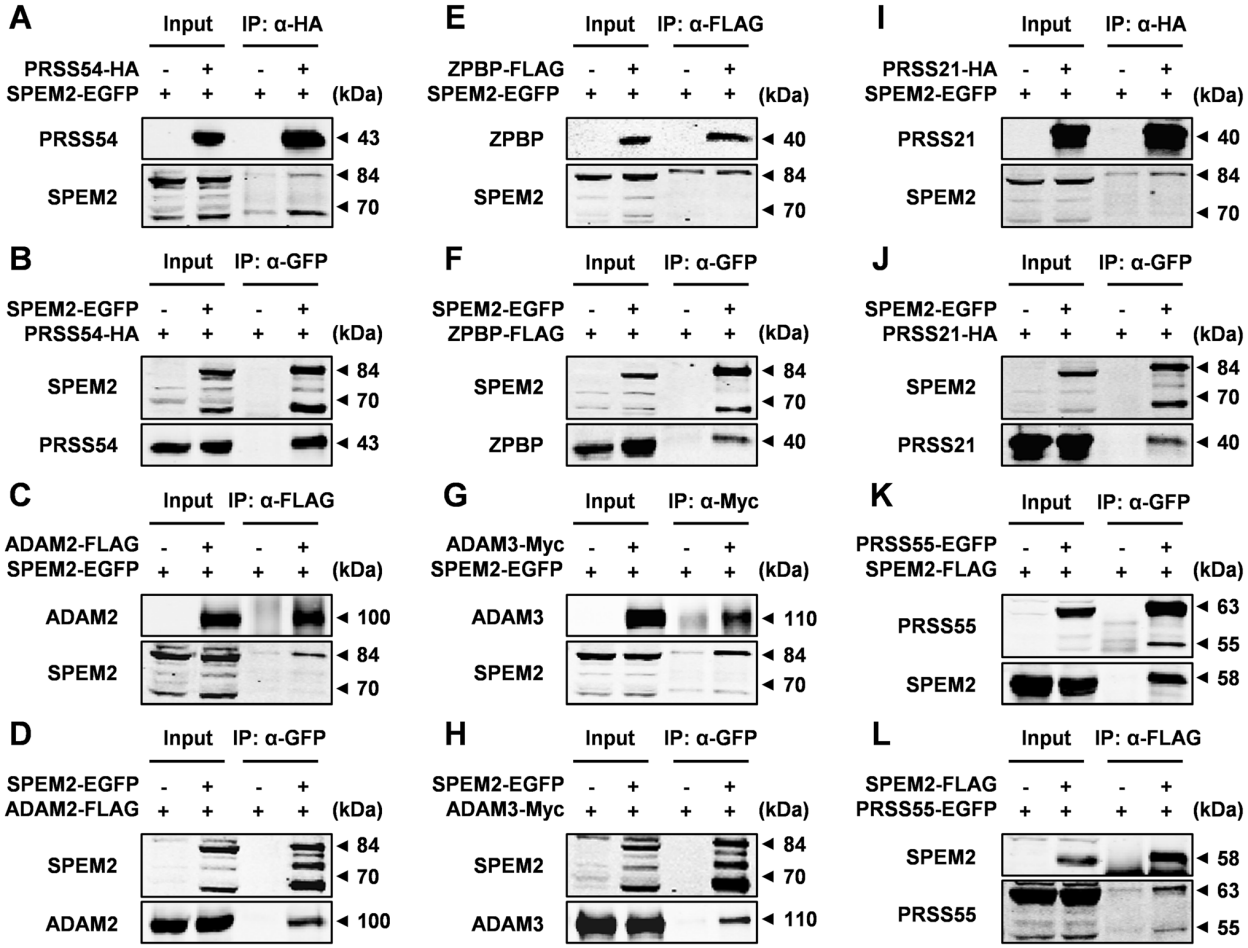
Interaction detection of SPEM2 with down-regulated protein PRSS54, ZPBP, PRSS21, ADAM2, ADAM3 or PRSS55 by in vitro Co-IP assays. **A–L** The interactions of EGFP-tagged SPEM2 (SPEM2-EGFP) or FLAG-tagged SPEM2 (SPEM2-FLAG) with either one of HA-tagged proteins (PRSS54-HA and PRSS21-HA) or FLAG-tagged proteins (ZPBP-FLAG and ADAM2-FLAG) or Myc-tagged ADAM3 (ADAM3-Myc) or EGFP-tagged PRSS55 (PRSS55-EGFP) in HEK293T cells were examined by Co-IP using either anti-HA (**A**, **I**) or anti-GFP (**B**, **D**, **F**, **H**, **J**, **K**) or anti-Myc (**G**) or anti-FLAG (**C**, **E**, **L**) mAb magnetic beads before analysis by western blotting with anti-GFP, anti-HA, anti-Myc, and anti-FLAG antibodies

To explore the potential mechanisms behind SPEM2 regulating sperm formation and function, we tried to examine the expression of a large number of key factors that are involved in regulating spermiogenesis and male fertility in testes and sperm by western blotting (Fig. 7A; Supplementary Fig. 5). Interestingly, 6 down-regulated proteins (ZPBP, PRSS21, PRSS54, PRSS55, ADAM2 and ADAM3) and 2 up-regulated proteins (PDILT and CLGN) were identified in *Spem2*-null sperm (Fig. 7A, B), while their expression levels were comparable in wt and KO testes (Fig. 7A). These dysregulated proteins mainly participate in acrosome development, sperm migration from the uterus into the oviduct, and sperm-oocyte interaction [18–25], providing a plausible explanation for the defects observed in *Spem2*-KO mice. We thus suggested that the male reproductive deficits of *Spem2*-KO mice might be because *Spem2* deficiency impairs the expression of those key molecules linked to spermiogenesis and fertilization. Of note, most of the down-regulated proteins are synthesized in the testis as a precursor protein which is processed and matured during sperm maturation in the epididymis. Significantly, ablation of *Spem2* led to down-regulation of their mature forms in epididymal sperm, while their precursor forms expressed in the testis were almost unaffected (Fig. 7A), indicating that SPEM2 might be involved in the regulation of processing and maturation of these proteins in the epididymal sperm. Accumulation of PDILT and CLGN, predominantly expressed in mouse testis, was found in *Spem2*-null sperm (Fig. 7A), potentially due to the presence of cytoplasmic remnants derived from late spermatid cytoplasm. STRING analysis revealed a complex interaction network among these dysregulated proteins (Fig. 7C). This finding prompted us to investigate whether there is any interaction between SPEM2 and these differential proteins. Co-IP and western blotting analyses indicated that SPEM2 exhibits detectable interactions with the down-regulated proteins ZPBP, PRSS21, PRSS54, PRSS55, ADAM2, and ADAM3 in vitro (Fig. 8A–L), which further emphasized the vital role of SPEM2 in spermiogenesis and fertilization. Meanwhile, no interaction between SPEM2 and the up-regulated proteins PDILT and CLGN was detected (Supplementary Fig. 6), which could serve as negative controls. These results thus strengthened our idea that SPEM2 might be involved in the regulation of sperm maturation by influencing the processing and maturation of their proteins. In addition, these new interactions provide a resource for future investigations of the molecular mechanisms of sperm development and maturation in mammals.

## Discussion

**Fig. 9:**
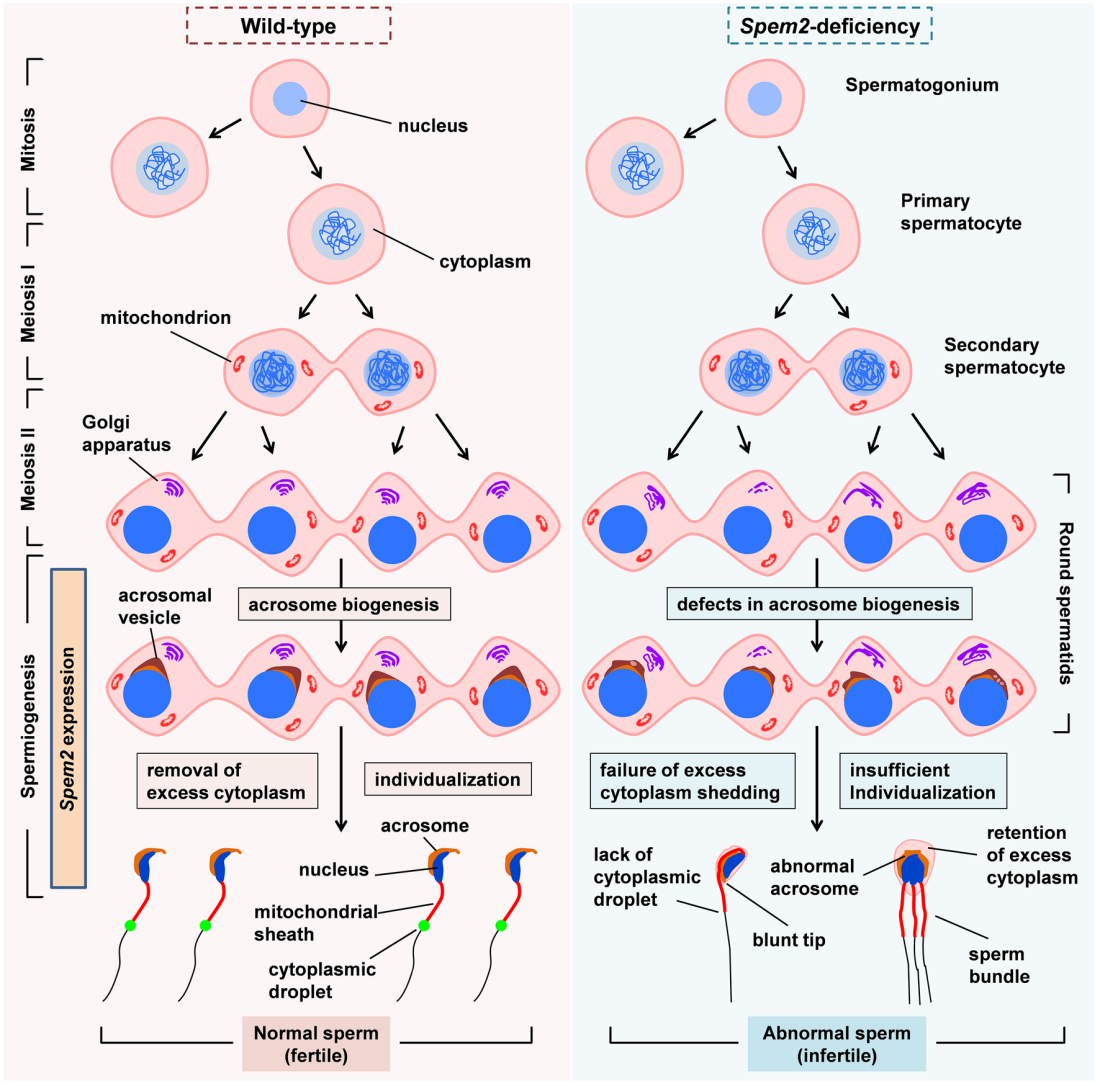
A diagrammatic summary showing the expression and role of *Spem2* in mouse testis. *Spem2* is specifically expressed during spermiogenesis. Consistent with its expression pattern in the mouse testis, *Spem2* deficiency leads to multiple abnormalities in spermiogenesis, mainly including abnormal acrosome biogenesis, failure of excess cytoplasm shedding, and insufficient spermatid individualization. No major defects in mitosis and meiosis are discovered. All these defects result in abnormalities in the sperm’s morphology and function and, therefore, give rise to male infertility

SPEM family molecules are highly enriched in human and mouse testis [3, 4], suggesting their potential functions in regulating testis development and spermatogenesis. A previous study revealed SPEM1’s indispensable role for the proper removal of cytoplasm in late spermiogenesis and genetic inactivation led to sperm deformation and male infertility [5], yet functional importance of other SPEM molecules in sperm development and male reproduction has been undefined. In the present study, we investigate the consequences of *Spem2* deficiency on sperm formation and function in mice. Compared with other testicular cells, *Spem2* is mainly expressed in postmeiotic germ cells, indicating its special role in the spermiogenesis. In line with the distribution pattern of this gene, ablation of *Spem2* in mice causes multiple abnormalities in spermiogenesis, including abnormal acrosome development, irregularly shaped nuclei, disrupted individualization, and inefficient removal of residual bodies and intercellular bridges (Fig. 9). All these defects result in abnormalities in the sperm’s morphology, motility and function, ultimately leading to male sterility.

*Spem2*-null sperm uniformly display failure of cytoplasmic removal, which closely resembles that of *Spem1* mutant sperm, suggesting SPEM family molecules might generally play a crucial role in the proper removal of cytoplasm in late spermiogenesis. Besides, disruption of spermatid individualization is a distinct feature observed in the testis of *Spem2*^−/−^ mice. Removal of excess cytoplasm and spermatid individualization are two key processes in spermiation [11], which are important for the generation of individual sperm with normal functions. In *Drosophila*, the cytoplasm removal and spermatid individualization have been extensively studied and numerous genes have been identified to be involved in these two processes [26]. Many of these genes have been characterized molecularly, and they have highlighted important mechanisms at play during these processes, including actin and microtubule dynamics, plasma membrane reorganization, and apoptotic elimination of cytoplasmic contents [26]. Spermatogenesis is strikingly similar in the mammals, and many molecular players are conserved between *Drosophila* and mammals [27, 28]. However, the regulatory mechanisms of sperm individualization and cytoplasm removal in *Drosophila* seem to differ from those in mammals. A typical case is myosin VI (MYO6), an actin-based molecular motor which plays important roles in spermiogenesis of various organisms [29]. In *Drosophila*, the MYO6 ortholog regulates actin reorganization during spermatid individualization [30], whereas in mice, this myosin is involved in maintaining the structural integrity of highly specialized actin-rich structures during acrosome biogenesis [31]. It can be seen that although this protein is conserved between *Drosophila* and mice, there are significant differences in its mode of action or protein function between the two species. In mice, only a limited number of genes have so far been identified to be involved in the processes of spermatid individualization and cytoplasm removal, including *Atg5*, *Atg7*, *Ct55*, *Arrdc5* and *DCUN1D1.* In humans, mutations in the *SPATA16* and *AURKC* genes cause the production of double-/multi-headed and multi-tailed sperm owing to an aborted cytokinesis [32–34]. Given that no major defects in meiosis were found among the *Spem2*^−/−^ spermatocytes, SPEM2 does not appear to have a role in cytokinesis during meiosis. Instead, SPEM2 mainly functions in the cytoplasmic removal and spermatid individualization during spermiogenesis. Little is currently known about the processes of spermatid individualization and cytoplasm removal during mammalian spermiogenesis. Therefore, it is particularly urgent and important to identify the genes that play key roles in the two processes and reveal their molecular mechanisms. *Spem2* mutant mice will be a useful model to study mammalian spermatid individualization and cytoplasm removal.

Recent studies suggest that autophagy may be a main player in the regulating of spermatid individualization and cytoplasm removal during mammalian spermiogenesis. Targeted inactivation of *Atg5*, *Atg7*, and *Ct55* resulted in a decrease in testicular autophagy activity, and KO mice with these genes displayed a similar phenotype as described here [35–37]. However, expression levels of ATG5 and ATG7 in *Spem2*^−/−^ mice are comparable to wt mice (Supplementary Fig. 5). Moreover, there is no significant difference in the expression of autophagosome marker LC3 and autophagy receptor SQSTM1 between the mutant and control mice (Supplementary Fig. 5). These results indicate that the autophagic flux is not disrupted in *Spem2*^−/−^ mice. The defects in sperm individualization and unnecessary cytoplasm removal have also been reported in other mouse models. Deletion of *DCUN1D1*, a component of the E3 for neddylation in mice results in retention of excess cytoplasm, failure of individualization and male infertility [38]. This suggests that protein neddylation is also required for mammalian sperm individualization and excess cytoplasm removal. Lack of *Spem2* did not affect the level of DCUN1D1 (Supplementary Fig. 5), indicating the abnormalities observed in *Spem2*^−/−^ mice might not be related to protein neddylation. In addition, ARRDC5, an arrestin protein, has been identified in the involvement of removal of excess cytoplasm and spermatid individualization during mouse spermiogenesis [39]. Arrestin molecules are known to function as modulators of protein ubiquitination [40], implying protein ubiquitination may contribute to the processes of spermatid individualization and cytoplasm removal. Interestingly, UBQLN1, which delivers poly-ubiquitinated proteins to proteasome for degradation, was reported as a SPEM1-interacting partner [41]. The interaction between SPEM1 and UBQLN1 suggests a role in the regulation of protein ubiquitination during spermiogenesis. Therefore, disruption of *Spem2* may cause aberrant ubiquitination, leading to redundant cytoplasm removal and insufficient sperm individualization.

During spermiation, the cytoplasm of elongated spermatids is phagocytozed by Sertoli cells [42], only a remnant of spermatid cytoplasm persists associated with the released spermatozoa, referred to as the cytoplasmic droplet (CD) [43]. In most species studied, there is convincing evidence that the CD migrates along the midpiece from neck to annulus during epididymal transit [44]. Noticeably, the retention of cytoplasmic droplet-like remnants in the head and neck region of *Spem2*-null sperm is associated with a lack of CDs, indicating that *Spem2* deficiency impairs the process of cytoplasm removal probably by preventing the CD from migrating from the head and neck region of the developing sperm. It has been suggested that CDs appear to play an important role in priming sperm for motility and fertility competence by serving as an energy source during epididymal sperm maturation [45]. Therefore, reduced sperm motility and reproductive inability in *Spem2*^−/−^ male mice may be associated with the loss of CDs. Moreover, retained cytoplasmic remnants obstruct the fusion of the plasma membrane and the outer acrosome membrane, thus resulting in impaired sperm acrosome reaction and fertilizing ability. In addition, several down-regulated proteins related to spermiogenesis and fertilization, including ZPBP, PRSS21, PRSS54, PRSS55, ADAM2 and ADAM3, have been identified in *Spem2*-null sperm. Intriguingly, most of these proteins are synthesized in the testis as a precursor protein which is processed and cleaved into mature form in epididymal sperm. Specially, ablation of *Spem2* leads to down-regulation of their mature forms in epididymal sperm, while their precursor forms in the testis are almost unaffected. In our Co-IP assays, all the six dysregulated proteins are detected to interact with SPEM2. This suggests that absence of *Spem2* may directly affect the processing and maturation of these proteins, which could further contribute to fertilization defects. These studies show that a combination of factors may be responsible for fertilization failure and male infertility in *Spem2*^−/−^ mice. Studies by Yan and colleagues have shown that SPEM1 may serve as a scaffold protein that can introduce ubiquitinated unwanted/defective proteins to UBQLN1, which then delivers these proteins to the proteasome for degradation [41]. Similarly, their studies have also shown that SPEM1 may function as a scaffold protein linking RANBP17’s cargo proteins to other processing machineries in the cytoplasm of elongating spermatids [46]. Given that SPEM2 does not contain any known protease domains, it may also function as a scaffold protein acting to deliver the precursor proteins to shuttle proteins, which then direct these protein precursors to the proteases for cleavage and maturation.

In conclusion, our findings highlight the important role of mouse *Spem2* in cytoplasmic removal, sperm individualization and fertilization. Further studies are warranted to elucidate the precise mechanisms by which SPEM2 regulates these processes and to determine the downstream targets and signaling pathways involved. Given that human *SPEM2* exhibits testis-enriched expression, and its function may be conserved in humans as well, it is a potential causative gene for human infertility, which makes genetic diagnosis possible in the future.

## Materials and methods

### Mice and genotyping

The *Spem2*-KO mice were generated by non-homologous recombination using CRISPR-Cas9-based gene targeting. Guide RNAs (gRNAs) and Cas9 mRNA were produced by T7 polymerase-driven in vitro transcription. The injection mix (50 ng/μL Cas9 mRNA and 50 ng/μL gRNAs) was injected into the cytoplasm of the fertilized eggs isolated from superovulated donor female C57BL/6 J mice. Surviving zygotes were subsequently implanted into the oviducts of pseudopregnant recipient females to obtain F0 generation. F0 generation were bred with normal C57BL/6J mice to produce F1 heterozygous mice. Heterozygous mice were intercrossed to produce the offspring with three different genotypes. The resulting pups were genotyped by PCR using primers P1, P2 and P3 (listed in Supplementary Table 2). Two bands, a 507-bp band as the wt allele and a 970-bp band as the mutant allele, were amplified by PCR. All mice were housed under specific pathogen-free conditions at a constant room temperature of 22–24 °C with a 12-h light/dark cycle, with free access to a diet of regular chow and water. All research protocols involving animal experiments were approved by the Institutional Animal Care and Use Committee of the Shanghai Research Center for Model Organisms.

### mRNA expression analyses

Total RNAs were extracted from multiple tissues of mice using TRIzol Plus RNA Purification Kit (Invitrogen, USA), and then 1 μg Total RNA was used to prepare cDNA using PrimeScript RT Master Mix (Takara, Japan) following manufacturer’s instructions. cDNAs were amplified using specific set of primers as listed in Supplementary Table 2 for RT-PCR or RT-qPCR analysis. RT-PCR products were separated by electrophoresis on 1.5% agarose gel and visualized by ethidium bromide staining. RT-qPCR was performed by Mastercycler ep realplex (Eppendorf, Germany) using SYBR Premix Ex Taq Kit (Takara, Japan). Resolution of the product of interest from nonspecific product amplification was achieved by melt curve analysis. Gene expression levels are normalized to beta actin (*Actb*) using 2^–ΔΔCt^ or 2^–ΔCt^ method.

### In situ hybridization

Adult mouse testes were fixed with Bouin’s buffer, embedded in paraffin, and sliced into 7 μm thick sections. DNA templates containing T7 or T3 RNA polymerase promoter site were generated by PCR reactions with specific set of primers as illustrated in Supplementary Table 2. They were then used for in vitro transcription using MAXIscript Kit (Thermo Fisher Scientific, USA) to synthesize single-stranded digoxigenin (DIG)-labeled RNA probes according to the manufacturer’s protocol. Hybridization was performed according to a previously reported protocol [47]. After hybridization and washing, the sections were incubated overnight with AP-coupled anti-DIG antibody at 4 °C. Color development reaction was performed at room temperature by incubating with BCIP/NBT substrate (Vector Laboratories, USA). After stopping the reaction, the sections were dehydrated, mounted, and then, observed under light microscope.

### Male fertility evaluation and sperm motility analysis

Male mice (8–12 weeks old, *n* = 5 each genotype) were each caged with two wt females for a month, during which plugged females were removed and replaced with new ones by checking copulatory plugs every day. Number of total females, plugged females, litters, and offspring was counted to calculate the frequency of copulatory plug (FCP), frequency of conception (FC), and litter size. Serum isolated from adult male mice (< 6 months old) was assayed for testosterone levels by use of a specific enzyme immunoassay kit (Sangon Biotech, China). Motility of sperm isolated from cauda epididymides of wt and *Spem2*^*−/−*^ mice was assessed as previously described [48].

### Histological and morphological analyses

Ratio of testis weights and body weight, hematoxylin and eosin (H&E) staining of testis and cauda epididymis paraffin sections as well as sperm smears, and peanut agglutinin (PNA) staining were all performed as previously described [49]. Different types of sperm were determined independently for each mouse by counting more than 200 sperm. The percentage of sperm with CD was determined on more than 200 sperm from three mice of each genotype. Sperm mitochondria were stained with MitoTracker Red CMXRos (Invitrogen, USA) according to the manufacturer’s instructions.

### Scanning and transmission electron microscopic analyses

Adult testes and cauda epididymal sperms were fixed in 2.5% glutaraldehyde/PBS buffer overnight at 4 °C. After washing in 1 × PBS, the testes were cut into small pieces of approximately 1 mm^3^, and postfixed in 1% OsO_4_/PBS buffer for 2 h at 4 °C. Then, the samples were dehydrated through a graded ethanol series. For scanning electron microscopic analysis, specimens were critical-point dried, briefly sputter-coated with gold particles, and observed with Nova NanoSEM 230 (FEI, Hillsboro, Oregon). For transmission electron microscopic analysis, samples were embedded with Epon812 and cut into ultrathin sections. Subsequently, the sections were stained with uranyl acetate and lead citrate. Images were taken using a Hitachi H-7650 TEM (Hitachi, Tokyo, Japan).

### Acrosome reaction analysis

Sperm isolated from cauda epididymides were incubated in TYH medium supplemented with 4 mg/ml BSA at 37 °C in a humidified incubator with 5% CO_2_ in air to allow capacitation. After 1 h, a small fraction of sperm was utilized to prepare the sperm smears. The remaining sperm were treated with calcium ionophore A23187, at a final concentration of 10 μM to induce acrosome reaction. After an additional 30-min incubation, sperm were spotted onto glass slides and air dried. Acrosome status was monitored by staining with fluorescein labeled PNA (Vector Laboratories, USA); only intact acrosomes could be stained by PNA, while acrosome-reacted sperm could not be stained by PNA [14]. The percentages of non-acrosome-reacted sperm were determined by counting more than 200 sperm. At least three independent experiments were carried out.

### Analysis of sperm fertilization ability

Sperm were released from the cauda epididymides of adult wt and *Spem2*^−/−^ mice. Oocytes were collected from the oviducts of wt females (3–4 weeks old) after pregnant mare serum gonadotropin (PMSG) and human chorionic gonadotropin (hCG) injection. In vivo and in vitro fertilization ability of wt and *Spem2*^−/−^ sperm were assessed as previously reported [20]. Sperm migration analysis was also performed as described previously [21, 23].

### Plasmid construction, cell culture and transfection

pEGFP-Spem2 vector was constructed by amplifying the entire open reading frame (ORF) of *Spem2* gene and cloned into the BglII/EcoRI sites of pEGFP-N2 vector (BD Biosciences, USA). pFLAG-Spem2 vector was constructed by amplifying its whole ORF and cloned into the HindIII/BglII sites of pFLAG-CMV-4 (Sigma-Aldrich, USA). pcDNA3.1-Prss21-HA vector was constructed by amplifying its ORF as well as HA sequence, and cloned into the HindIII/EcoRI sites of pcDNA3.1 ( +) vector (Invitrogen, USA). The eukaryotic expression plasmids including pEGFP-Prss55, pCAG-Adam2-FLAG, pCAG-Clgn-FLAG, pCAG-Pdilt-FLAG, pCAG-Adam3-Myc, pCAG-Prss54-HA and pCAG-Zpbp-FLAG have been reported before [20, 21, 50]. The HEK293T cells, which were originally purchased from ATCC (Manassas, VA, USA) and maintained in our laboratory, were cultured in high glucose DMEM medium (Gibco) containing 10% FBS at 37 °C with 5% CO_2_. The recombinant plasmids were transfected into cells using Lipofectamine 3000 transfection reagent (Invitrogen). Cells were harvested 48 h after transfection and used for subsequent analysis of the interaction of SPEM2 and other proteins.

### Immunofluorescence staining

For sperm immunofluorescence, sperm smears were acquired by spotting sperm isolated from cauda epididymis onto poly-L-lysine coated glass slides, which were then air dried and fixed with 4% PFA. The slides were permeabilized with 0.1% Triton X-100 and washed with PBS three times. After blocking with 5% normal goat serum, slides were incubated with primary antibodies (listed in Supplementary Table 3) at 4 °C overnight, followed by staining with Alexa Fluor 488-conjugated secondary antibody (Invitrogen) at room temperature for 1 h. After washing with PBS, the slides were mounted with fluorescence mounting medium (DAKO, Glostrup, Denmark), coverslipped and examined under a Nikon Eclipse 80i microscope (Nikon, Tokyo, Japan) or LSM 880 microscope (Zeiss, Oberkochen, Germany). For subcellular localization of SPEM2 protein, 1 × 10^5^ cells were plated on coverslips in 35-mm dish, transfected with pEGFP-N2 or pEGFP-Spem2 vector, and cultured in high glucose DMEM medium (Gibco) containing 10% fetal bovine serum (FBS) for 24 h. The cells were then stained with Golgi-Tracker Red (Beyotime Biotechnology, China) according to the manufacturer's protocol. The EGFP-tagged proteins and stained Golgi apparatus were visualized under a Laser Confocal Microscope (Zeiss).

### Western blotting and co-immunoprecipitation

Tissue or cell samples were lysed for 30 min in cold RIPA buffer (Beyotime Biotechnology, China) supplemented with protease and phosphatase inhibitor cocktails (Roche, Switzerland). After centrifugation at 12,000 rpm for 20 min, the supernatant was harvested for further analysis. Proteins of mature sperm collected from the cauda epididymides of mice were extracted by 1 mM HCl containing 1% SDS (HCl/SDS) added with protease and phosphatase inhibitor cocktails at room temperature for 1 h. After centrifugation at 16,000 g for 10 min, the supernatants were harvested as sperm protein extracts. Protein concentration was determined with a BCA protein assay kit (Pierce, USA). Equal amounts of protein samples were separated by SDS-PAGE under reducing conditions and transferred onto nitrocellulose membranes. After blocking with 5% skimmed milk, membranes were incubated with primary antibodies (listed in Supplementary Table 3) overnight at 4 °C and subsequently incubated with secondary antibodies conjugated with IRdye 800CW (LI-COR, USA). Membranes were scanned by Odyssey Infrared Imager (LI-COR, USA). Densitometry was calculated with Image J software.

For co-immunoprecipitation (Co-IP) analysis in HEK293T cells, NETN lysis buffer (20 mM Tris–HCl pH 7.5, 100 mM NaCl, 6 mM EDTA, 0.5% NP-40, and protease and phosphatase inhibitor cocktails) was used to extract proteins. 1 mg of proteins was incubated with 50 μl of anti-HA, anti-GFP, anti-FLAG or anti-Myc mAb magnetic beads (MBL, Japan) overnight at 4 °C. The magnetic beads were separated by the magnetic separator, washed with lysis buffer for five times, eluted with 1 × SDS sample buffer and analyzed by western blotting.

### Bioinformatics analyses

SPEM2 protein sequences of multiple mammalian species were downloaded from the UniProt Knowledgebase (UniProtKB) (https://www.uniprot.org/). Sequence alignment and distance analyses of mouse, rat, rabbit, pig, chimpanzee and human SPEM2 proteins were performed using the MegAlign program (DNASTAR, Madison, WI). Phylogenetic trees of SPEM2 in mammalian species were constructed by neighbor-joining method using Molecular Evolutionary Genetics Analysis (MEGA) software (https://www.megasoftware.net/). The functional domain prediction about SPEM2 amino acid sequences was analyzed by InterPro (https://www.ebi.ac.uk/interpro/). The gene symbols corresponding to differentially expressed proteins identified in *Spem2-*KO mice were sent to the Search Tool for the Retrieval of Interacting Genes/Proteins (STRING version 12.0 [51]; https://cn.string-db.org/), to build a network using edge information from 3 separate form of evidence: databases, experiments, and text mining. We used 0.4 (medium confidence) as the value for the edge confidence provided by STRING.

## Statistical analysis

All data are presented as mean ± standard error of mean (SEM) (*n* ≥ 3), unless otherwise stated. Statistical significance between two groups was analyzed using a two-tailed unpaired Student’s *t* test. For all the statistical tests, *P* values less than 0.05 were considered statistically significant.

### Supplementary Information

Below is the link to the electronic supplementary material.

Supplementary file1 (DOCX 15001 KB)

## Data Availability

The data underlying this article are available in the article and in its online supplementary material.
