# Supplementary material for: Spem2, a novel testis-enriched gene, is required for spermiogenesis and fertilization in mice
Source: Cell Mol Life Sci. 2024 Feb 29;81(1):108. doi: 10.1007/s00018-024-05147-w (PMC10904452; doi:10.1007/s00018-024-05147-w)
Supplement: Supplementary file 1 — Supplementary file1 (DOCX 15001 KB) [file 18_2024_5147_MOESM1_ESM.docx]

**Supplementary Information**

**
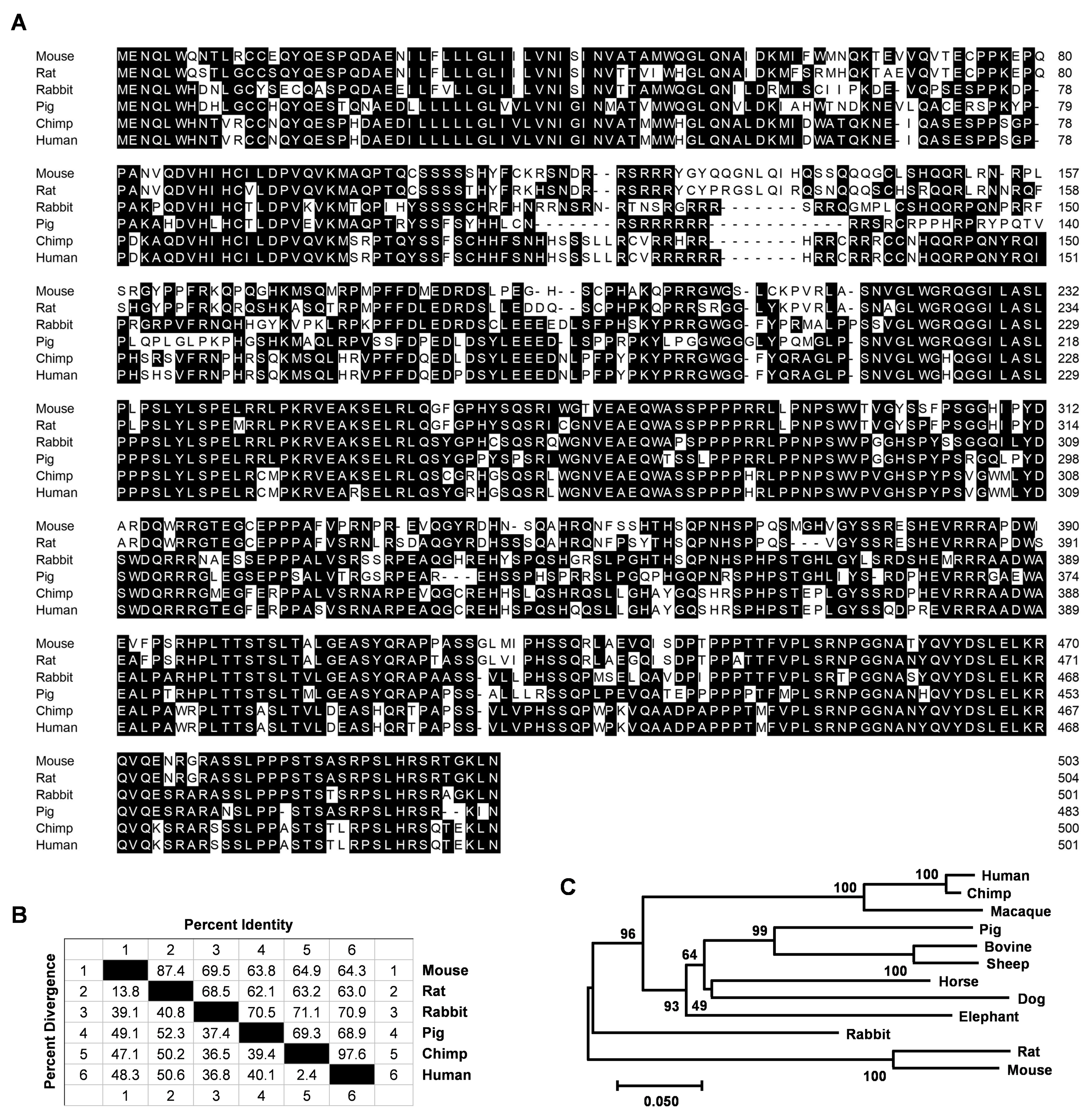
**

**Supplementary Fig. 1** Evolutionary conservation of SPEM2 in mammalian species. **A** Alignment analyses of mouse, rat, rabbit, pig, chimpanzee, and human SPEM2 proteins. Conserved residues are filled. **B** Sequence distances of six mammalian SPEM2 orthologs. **C** Phylogenetic trees of SPEM2 in twelve mammalian species. The numbers in the dendrogram are bootstrap value (%).

**
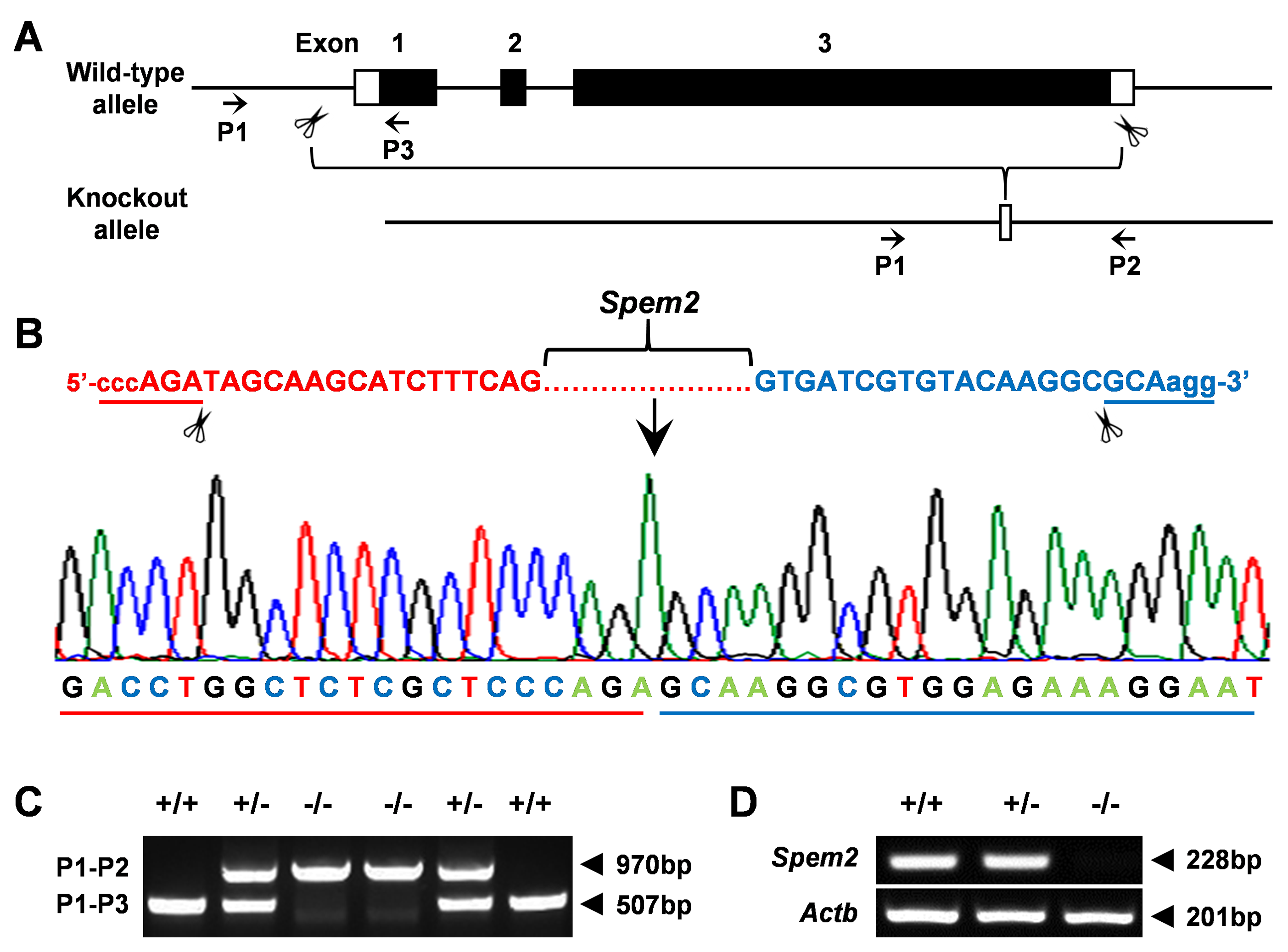
**

**Supplementary Fig. 2** Generation of *Spem2*-KO mice. **A** Schematic diagram of the targeting strategy of *Spem2* using CRISPR/Cas9. P1-P3 indicate genotyping primers and their relative positions. **B** Sequencing analysis of the mutant mouse line. gRNA1 sequence (red capitalized bases), gRNA2 sequence (blue capitalized bases), and their respective protospacer adjacent motif (PAM) sequence (3 colored lowercase bases) are indicated. **C** Genotyping of *Spem2* mutant mice by PCR. **D** Expression of *Spem2* in the testes of wt (+/+), heterozygous (+/-) and homozygous (-/-) mice was examined by RT-PCR. *Actb* served as a loading control.

**
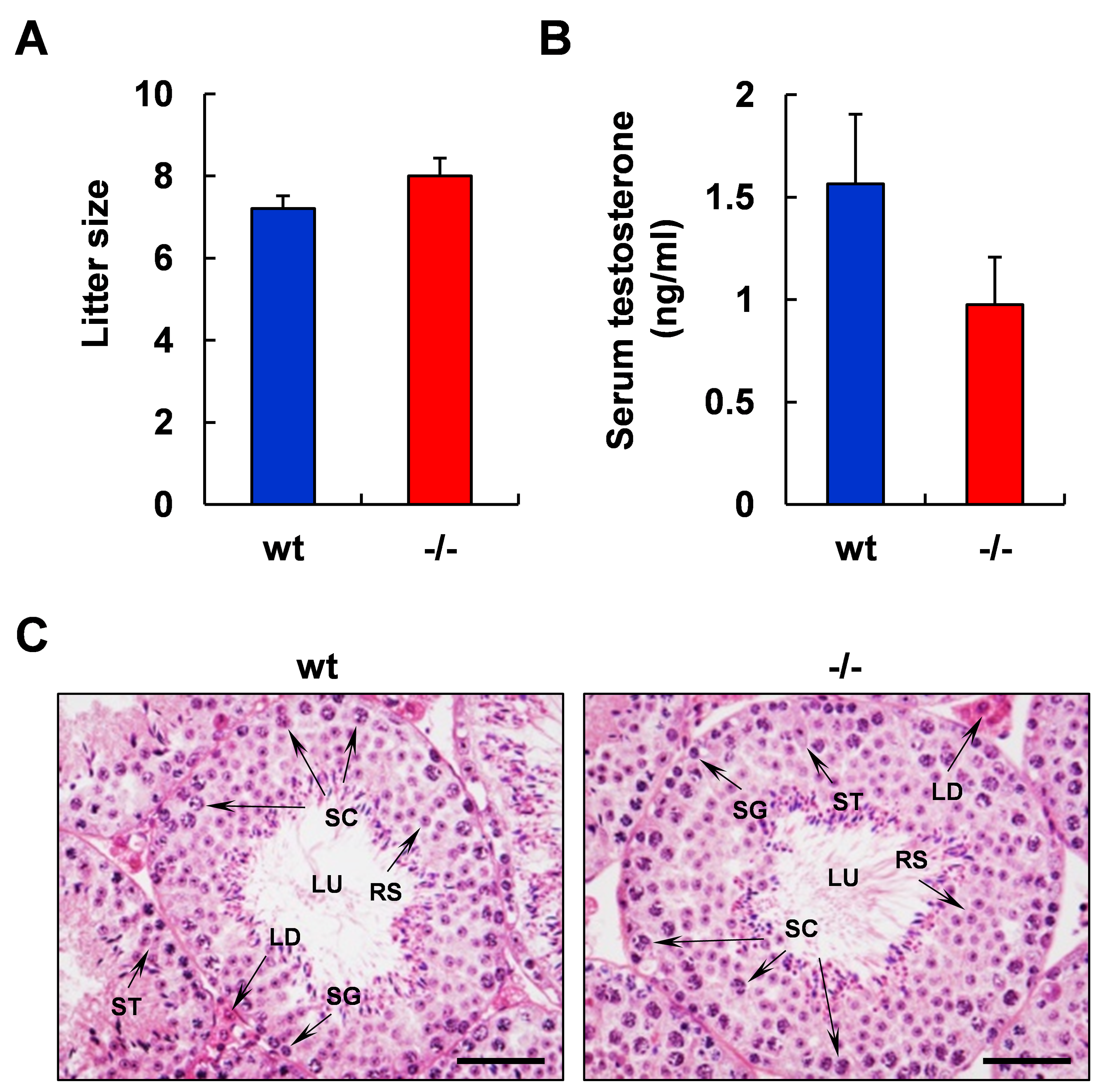
**

**Supplementary Fig. 3** Assessment on the influence of *Spem2* deficiency on female fertility, serum testosterone level and spermatogenesis. **A** wt and *Spem2*^−/−^ female mice were bred with wt male mice, and the litter sizes were measured. The fertility of KO females was not different compared with wt control. Data bars are mean ± SEM. **B** The concentration of the serum testosterone was measured by extracting the eyeball blood from wt and *Spem2*^−/−^ male mice. There was no significant difference in serum testosterone level between wt and *Spem2*^−/−^ males (*P* > 0.05). Data bars are mean ± SEM. **C** H&E staining sections of testes from wt and *Spem2*^−/−^ mice, showing intact seminiferous tubules and epithelium with seemingly normal spermatogenesis in *Spem2*^−/−^ mice, including properly organized layers of spermatogonia (SG), spermatocytes (SC), and round spermatids (RS) and differences compared to testes from wt mice were not observable. ST, Sertoli cells; LD, Leydig cells; LU, lumen. Scale bar, 50 μm.

**
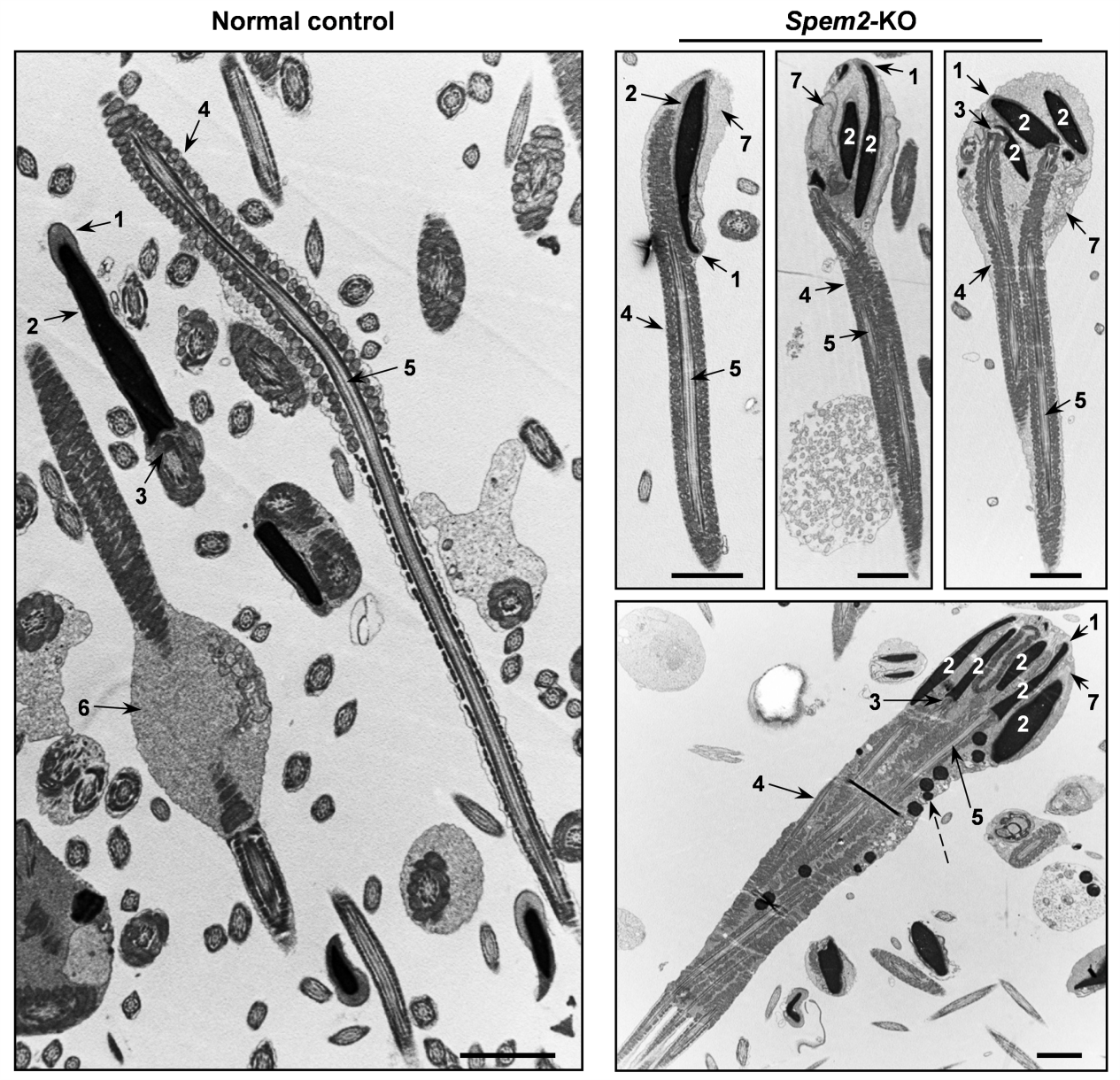
**

**Supplementary Fig. 4** TEM analyses for epididymal sperm of normal control and *Spem2*-KO mice. 1, acrosome; 2, nucleus; 3, neck; 4, mitochondrial sheath; 5, axoneme with typical “9+2” microtubule structure (9 pairs of peripheral and two central microtubules); 6, cytoplasmic droplet; 7, cytoplasmic remnant. The dashed arrow may represent lipid droplets and RNAs (leftover ribosomes). Scale bar, 2 μm.

**
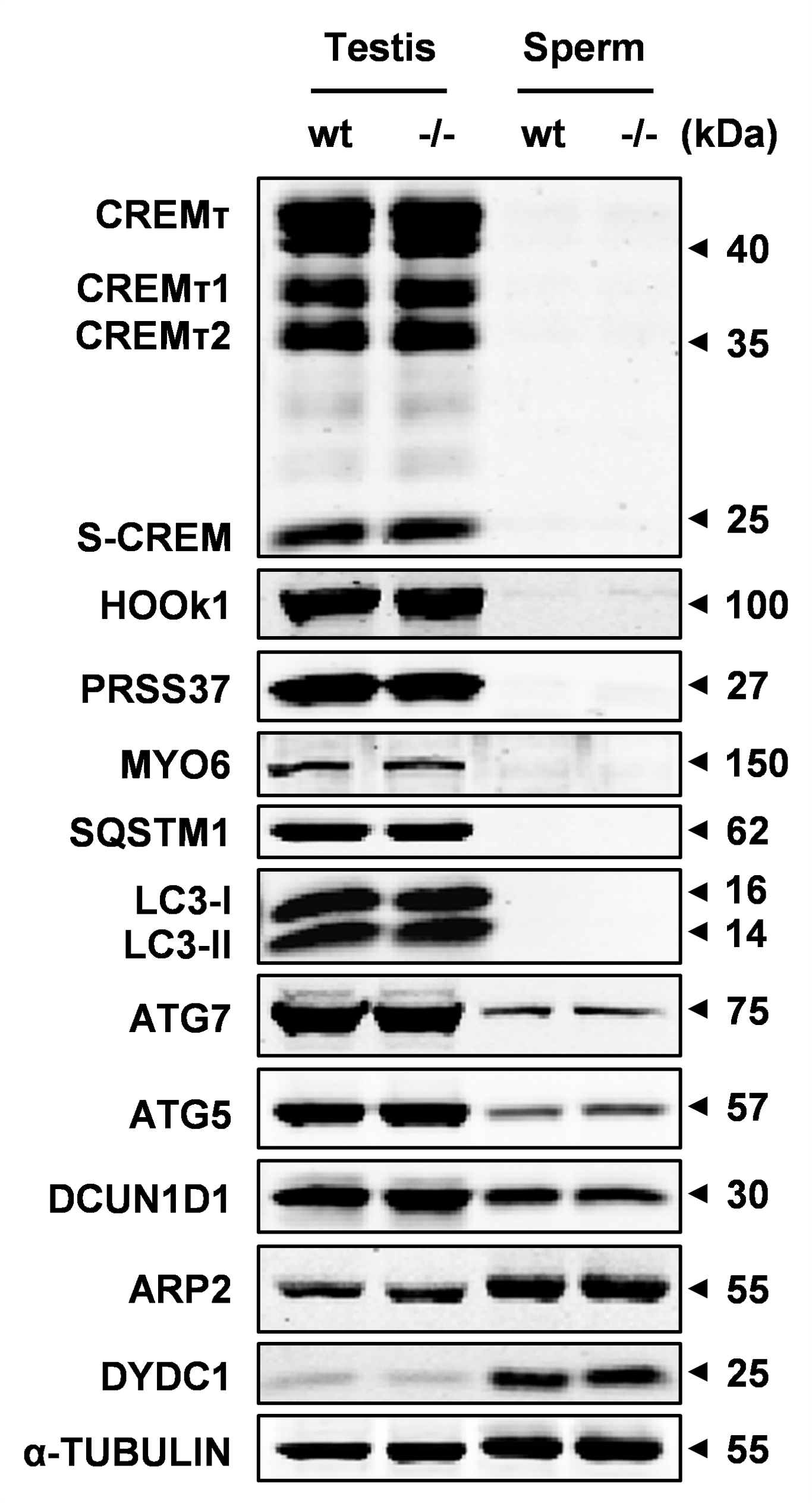
**

**Supplementary Fig. 5** Western blotting analysis of a large number of proteins related to spermiogenesis and male reproduction in the testis and epididymal sperm of adult wt and *Spem2*^-/-^ mice. α-TUBULIN was used as a loading control.

**
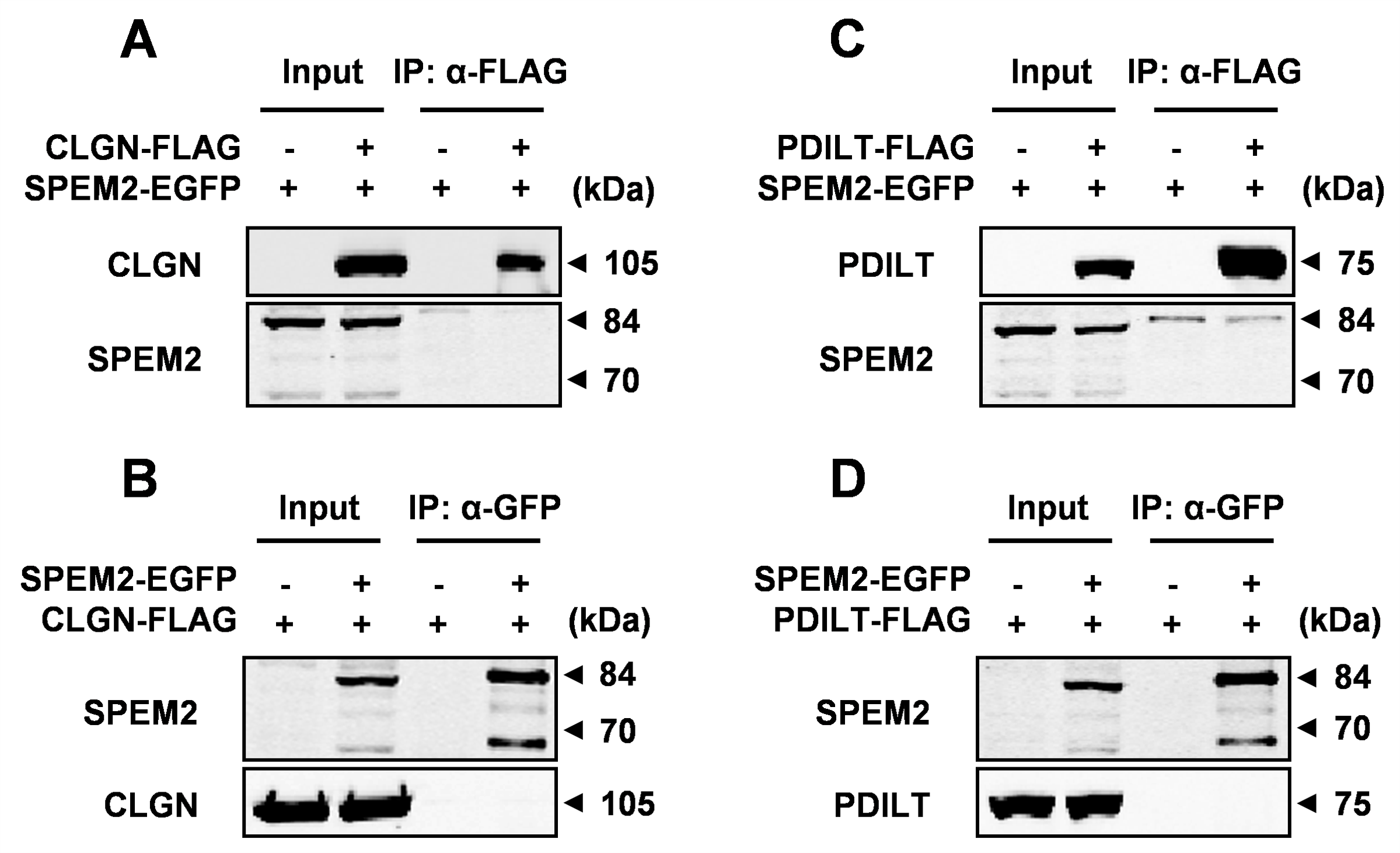
**

**Supplementary Fig. 6** No interaction between SPEM2 and up-regulated protein CLGN or PDILT was detected *in vitro*. **A-D** Co-IP analysis of EGFP-tagged SPEM2 (SPEM2-EGFP) and FLAG-tagged proteins (CLGN-FLAG and PDILT-FLAG) in HEK293T cells.

**Supplementary Table 1** Abnormal sperm motility by CASA analysis in *Spem2*^-/-^ mice

| CASA parameters | wt  (n=5) | *Spem2*^-/-^  (n=5) |
| --- | --- | --- |
| Total motility (%) | 59.0 ± 1.4 | 48.5 ± 1.8*** |
| Progressive motility (%) | 28.8 ± 0.8 | 25.4 ± 2.2 |
| Rapid motility (%) | 44.4 ± 1.2 | 34.3 ± 2.4** |
| Static cell (%) | 41.0 ± 1.4 | 51.5 ± 1.8*** |
| Path velocity (μm/sec) | 77.4 ± 1.9 | 82.5 ± 3.6 |
| Prog. velocity (μm/sec) | 44.3 ± 1.2 | 50.4 ± 2.8 |
| Track speed (μm/sec) | 150.1 ± 3.9 | 159.8 ± 6.7 |
| Lateral amplitude (μm) | 11.0 ± 0.3 | 13.6 ± 0.5*** |
| Beat frequency (Hz) | 27.2 ± 0.6 | 24.8 ± 1.4 |
| Straightness (%) | 54.5 ± 0.8 | 58.3 ± 0.8** |
| Linearity (%) | 29.9 ± 0.5 | 31.4 ± 0.5 |
| Elongation (%) | 50.2 ± 0.8 | 49.2 ± 1.2 |
| Area (μm sq) | 6.4 ± 0.2 | 8.5 ± 0.4*** |

*Two-sided Student’s *t* test. ***p* < 0.01; ****p* < 0.001.

**Supplementary Table 2** Primers used in this study

| Designation | Forward (5’*-*3’) | Reverse (5’*-*3’) |
| --- | --- | --- |
| RT-PCR/RT-qPCR |  |  |
| *Spem2* | CATGGAAAACCAGCTGTGGC | GGGGGACATTCGGTAACCTG |
| *Actb* | GGCTCCTAGCACCATGAAGA | AGGGTGTAAAACGCAGCTCAG |
| *SPEM2* | ACGTGGCAACTATGATGTGGC | ATCTTCACCTGCACAGGGTCC |
| *ACTB* | AGGATTCCTATGTGGGCGAC | GGTCTCAAACATGATCTGGGT |
| *In situ* hybridization | AATTAACCCTCACTAAAGGGAGAcctccctctacctgtcacca | TAATACGACTCACTATAGGGAGAgtggggccctttggtatgaa |
| Genotyping |  |  |
| P1 | GGGCGCTGGTGCTAGAGGGATTAT |  |
| P2 |  | GGGTGCATTGAGACCTCCGATCTG |
| P3 |  | GTGTTCTGCCACAGCTGGTTTTCC |
| Plasmid constructs |  |  |
| pEGFP-Spem2 | agcgctaccggactcagatctGCCACCATGGAAAACCAGCTG | gtaccgtcgactgcagaattcGTTAAGTTTCCCTGTCCGGCT |
| pFLAG-Spem2 | aaagacgatgacgacaagcttATGGAAAACCAGCTGTGGCA | actggtaccgatatcagatctTCAGTTAAGTTTCCCTGTCCGG |
| pcDNA3.1-Prss21-HA | TATAAGCTTGCCACCATGGGCGCGCGGGGCAAGACGTTG | ATAGAATTCTCAAGCGTAATCTGGAACATCGTATGGGTAGGCAGGCCTCAGCAAAGAGGAAGCCCAGGC |

**Supplementary Table 3** Antibodys employed in this study

| Antibody | Supplier | Catologue | Dilution |
| --- | --- | --- | --- |
| GFP-Tag Rabbit pAb | Abcam | ab6556 | WB (1:1000) |
| FlAG-Tag Mouse mAb | Sigma-Aldrich | F3165-1MG | WB (1:1000) |
| FlAG-Tag Rabbit mAb | Cell Signaling Technology | 14793 | WB (1:1000) |
| 15-LOX Mouse mAb | Santa Cruz Biotechnology | sc-133085 | IF (1:50) |
| CREM Mouse mAb | Sigma-Aldrich | WH0001390M2 | WB (1:1000) |
| HOOK1 Rabbit mAb | Abcam | ab150397 | WB (1:1000) |
| PRSS37 Rabbit pAb | Sigma-Aldrich | HPA020541 | WB (1:1000) |
| DYDC1 Goat pAb | Sigma-Aldrich | SAB2501402 | WB (1:1000) |
| MYO6 Rabbit pAb | ABclonal | A13033 | WB (1:1000) |
| SQSTM1/p62 Rabbit mAb | ABclonal | A19700 | WB (1:1000) |
| LC3B Rabbit mAb | Abcam | ab192890 | WB (1:1000) |
| ATG7 Rabbit mAb | ABclonal | A19604 | WB (1:1000) |
| ATG5 Rabbit mAb | ABclonal | A19677 | WB (1:1000) |
| ZPBP Mouse pAb | In house | / | WB (1:500) |
| ARP2 Rabbit pAb | ABclonal | A5734 | WB (1:1000) |
| α-Tubulin Mouse mAb | Sigma-Aldrich | 05829 | WB (1:1000) |
| PRSS54 Mouse mAb | Aviva Systems Biology | MA43587-003 | WB (1:1000) |
| PRSS21 Goat pAb | R&D Systems | AF6820 | WB (1:500) |
| ADAM3 Mouse pAb | Santa Cruz Biotechnology | sc-365288 | WB (1:1000) |
| ADAM2 Mouse pAb | Millipore | MAB19292 | WB (1:1000) |
| PRSS55 Mouse mAb | Aviva Systems Biology | MA43587-005 | WB (1:1000) |
| PDILT Rabbit pAb | Abcam | ab191851 | WB (1:1000) |
| CLGN Rabbit mAb | Abcam | ab171971 | WB (1:1000) |
| DCUN1D1 Rabbit mAb | Abcam | ab181233 | WB (1:1000) |
| HA-Tag Rabbit pAb | Abcam | ab9110 | WB (1:1000) |
| Myc-Tag Mouse mAb | Santa Cruz Biotechnology | sc-40 | WB (1:1000) |
